# Two-Dose Ceftiofur Treatment Increases Cephamycinase Gene Quantities and Fecal Microbiome Diversity in Dairy Cows Diagnosed with Metritis

**DOI:** 10.3390/microorganisms11112728

**Published:** 2023-11-08

**Authors:** Claudia Ossa-Trujillo, Ethan A. Taylor, Fatima Sarwar, Javier Vinasco, Ellen R. Jordan, Jose A. García Buitrago, G. Robert Hagevoort, Sara D. Lawhon, Juan M. Piñeiro, Jessica Galloway-Peña, Keri N. Norman, Harvey Morgan Scott

**Affiliations:** 1 Department of Veterinary Integrative Biosciences, College of Veterinary Medicine and Biomedical Sciences, Texas A&M University, College Station, TX 77843, USA; cossat@tamu.edu; 2 Department of Veterinary Pathobiology, College of Veterinary Medicine and Biomedical Sciences Texas A&M University, College Station, TX 77843, USA; eataylor2136@gmail.com (E.A.T.); fatimasarwar15@gmail.com (F.S.); jvinasco-torres@cvm.tamu.edu (J.V.); slawhon@cvm.tamu.edu (S.D.L.); jgallowaypena@cvm.tamu.edu (J.G.-P.); 3 Department of Animal Science, Texas A&M University, Dallas, TX 75252, USA; txjordanqh@yahoo.com; 4 Department of Extension Animal Sciences and Natural Resources, New Mexico State University, Clovis, NM 88101, USA; jarmando@nmsu.edu (J.A.G.B.); dairydoc@nmsu.edu (G.R.H.); 5 Department of Animal Science, Texas A&M University, Amarillo, TX 79106, USA; juan.pineiro@ag.tamu.edu

**Keywords:** two-dose CCFA, 3rd generation cephalosporin, fluoroquinolone, macrolide, antibiotic resistance genes, dairy cattle, metritis, fecal microbiota, metagenomics, ecological diversity

## Abstract

Antimicrobial resistance is a significant concern worldwide; meanwhile, the impact of 3rd generation cephalosporin (3GC) antibiotics on the microbial communities of cattle and resistance within these communities is largely unknown. The objectives of this study were to determine the effects of two-dose ceftiofur crystalline-free acid (2-CCFA) treatment on the fecal microbiota and on the quantities of second-and third-generation cephalosporin, fluoroquinolone, and macrolide resistance genes in Holstein-Friesian dairy cows in the southwestern United States. Across three dairy farms, 124 matched pairs of cows were enrolled in a longitudinal study. Following the product label regimen, CCFA was administered on days 0 and 3 to cows diagnosed with postpartum metritis. Healthy cows were pair-matched based on lactation number and calving date. Fecal samples were collected on days 0, 6, and 16 and pooled in groups of 4 (*n* = 192) by farm, day, and treatment group for community DNA extraction. The characterization of community DNA included real-time PCR (qPCR) to quantify the following antibiotic resistance genes: *bla*_CMY-2_, *bla*_CTX-M_, *mphA*, *qnrB19,* and the highly conserved 16S rRNA back-calculated to gene copies per gram of feces. Additionally, 16S rRNA amplicon sequencing and metagenomics analyses were used to determine differences in bacterial community composition by treatment, day, and farm. Overall, *bla*_CMY-2_ gene copies per gram of feces increased significantly (*p* ≤ 0.05) in the treated group compared to the untreated group on day 6 and remained elevated on day 16. However, *bla*_CTX-M_*, mphA,* and *qnrB19* gene quantities did not differ significantly (*p* ≥ 0.05) between treatment groups, days, or farms, suggesting a cephamycinase-specific enhancement in cows on these farms. Perhaps unexpectedly, 16S rRNA amplicon metagenomic analyses showed that the fecal bacterial communities from treated animals on day 6 had significantly greater (*p* ≤ 0.05) alpha and beta diversity than the untreated group. Two-dose ceftiofur treatment in dairy cows with metritis elevates cephamycinase gene quantities among all fecal bacteria while paradoxically increasing microbial diversity.

## 1. Introduction

Antibiotics have improved human health outcomes since their discovery in 1928 [1]; however, antimicrobial resistance (AMR) has expanded, leading to worldwide public health concerns [2]. In the United States, human AMR infections reach 2.8 million cases annually, with more than 35,000 deaths, according to the Centers for Disease Control and Prevention (CDC) [3], and it has been estimated that AMR infections will lead to 10 million worldwide deaths annually by 2050 [4]. Since 2000, there has been an increase in resistant infections such as those caused by extended-spectrum β-lactamase (ESBL)-producing Enterobacteriaceae. Among ESBLs, CTX-M-type enzymes are the most diverse and commonly recognized. [5,6].

*Escherichia coli* is one of six principal pathogens responsible for AMR-associated deaths worldwide [7]. *E. coli* is a gram-negative coliform bacterium within the Enterobacteriaceae family and represents an essential part of the normal gut microbiota of warm-blooded organisms, such as birds and mammals [8,9]. *E. coli*, among the other members of the intestinal microbial community, plays an essential function in host-pathogen interaction and may contribute to severe infections under opportunistic conditions [10]. *E. coli* can potentially acquire resistance genes due to selection pressures, such as antibiotic administration, predominantly through horizontal gene transfer between bacteria [9,11]. Subsequently, *E. coli* replicates in the intestinal microbiota and may act as a reservoir for spreading multidrug-resistant (MDR) genotypes and plasmid types [12]. In addition to ESBL, other resistant genotypes of importance in *E. coli* include 2nd and 3rd generation cephalosporins (3GC), such as cephamycinase (e.g., *bla*_CMY-2_), fluoroquinolone (e.g., *qnrB*), and macrolide (e.g., *mphA*) [13,14,15,16].

β-lactams are the most commonly administered bactericidal antibiotics in the U.S. (accounting for 65% of the antibiotic market) in human and veterinary medicine due to their broad spectrum of activity against gram-negative and gram-positive bacteria [17]. One of the mechanisms of action for β-lactams is to target peptidoglycan synthesis by adhering to the penicillin-binding protein (PBP) enzyme to inhibit bacterial cell wall synthesis [18]. Therefore, β-lactams affect bacterial community dynamics and create dysbiosis in the gut microbiota by killing off large numbers of susceptible bacteria [19].

Ceftiofur crystalline free acid (CCFA) is a 3GC within the β-lactam class of antibiotics, approved for use in veterinary medicine, and is the preferred antibiotic to treat acute metritis in dairy cows because the administration does not require milk withholding [20]. Metritis causes significant economic loss for the dairy industry in the United States due to reduced milk production, reproduction, and herd survival; meanwhile, low-producing animals are 30% more likely to be culled than their herd mates [21]. Previous studies have demonstrated that the administration of CCFA in cattle elevates the levels of 3GC-resistant enteric *E. coli* at the first eligible slaughter date (13 days after CCFA administration) [22]. Food products contaminated with resistant bacteria increase the risk of AMR bacteria entering the food chain and subsequently being transmitted to humans [23].

The use of fluoroquinolones and macrolides is prohibited in dairy cattle over 20 months of age [24,25]; however, studies have reported *qnrB* and *mphA* resistance genes in *E. coli* isolates from the feces of adult dairy cattle [26]. We hypothesize that 3GC antibiotics may co-select for macrolides and fluoroquinolone genes as they can be co-located on plasmids. Notably, 3GC, along with fluoroquinolone and macrolide classes, are considered the highest priority/critically important antibiotic classes (HP-CIA) for human medicine by the World Health Organization (WHO) [27,28]. Hence, the objectives of this study were to determine the effects of two-dose ceftiofur crystalline-free acid on the fecal microbiota and the quantities of third-generation cephalosporin (3GC), fluoroquinolone (FQ), and macrolide (ML) resistance genes present in fecal samples from Holstein-Friesian dairy cows, with and without a diagnosis of metritis, in the southwestern United States.

## 2. Materials and Methods

### 2.1. Ethical Statement

All animal experiments and animal care conditions were approved by the Texas A&M University Institutional Animal Care and Use Committee (Animal Use Protocol (AUP) No. 2016-0183).

### 2.2. Study Design

**Figure 1:**
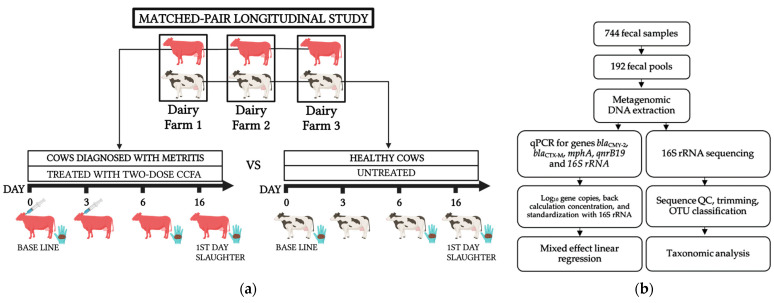
Overview of the experimental design. (**a**) Schematic description of the matched-pair longitudinal study design (created with BioRender.com accessed on 1 September 2023) and (**b**) Overall methods workflow.

The experimental design has been previously described in detail [22]. Briefly, we conducted a pair-matched longitudinal study of 248 cattle across two seasons (spring/summer and autumn/winter) from April 2017 to April 2018 at three large U.S. southwestern dairy farms. We sampled two groups of Holstein-Friesian dairy cows on each farm; one group was those cows diagnosed with metritis and therefore treated with 2-CCFA. Each treated cow was matched by lactation number with a healthy cow (specifically, absent of metritis and untreated) on the same farm (Figure 1a) and having calved at nearly the same time/date. Healthy animals were not treated due to the U.S. FDA regulation (21 CFR Part 530), which has prohibited the off-label usage of ceftiofur in food-producing animals since April 2012 [29]. Negative control (i.e., untreated) cows diagnosed with metritis were not permitted per the AUP nor by the owners (farmers) of the animals.

### 2.3. Animal Sample Collection and Sample Processing

Fecal samples were collected per rectum with an obstetric sleeve from each set of pair-matched cows on day 0 to represent the pre-treatment baseline. The first dose of CCFA was administered, following label instructions, subcutaneously at the base of the ear to cows diagnosed with metritis after collecting the first fecal sample, and the second dose was administered 72 h later. Additional fecal samples were collected from pair-matched cows on day 6 (expected peak antibiotic effect) after antibiotic treatment and day 16 (first-eligible slaughter day following treatment) (Figure 1a). Samples were shipped to the laboratory at Texas A&M University, College Station, TX, USA. Fecal samples were stored in two tubes (one mixed with 50% glycerol in a 1:1 ratio and the other without glycerol) and preserved at −80 °C.

### 2.4. Quantitative Real-Time PCR

#### 2.4.1. Total Community DNA Extraction

In total, 192 fecal pools were prepared by mixing one gram of each of 4 non-glycerol samples matched on treatment group (2-CCFA or no treatment), farm (1, 2 or 3), and day (0, 6, or 16) (Table S1). According to the manufacturer’s protocol, 250 mg of pooled feces was used for total community DNA extraction using the DNeasy PowerSoil Kit (Qiagen, Valencia, CA, USA) in the QIAcube system (Qiagen, Valencia, CA, USA). The quality (260/280 nm ratio) and quantity (ng/μL) of the community DNA were measured via the FLUOstar Omega LVis Plate (BMG LabTech Inc., Cary, NC, USA). DNA samples were stored at −30 °C for further molecular analyses (Figure 1b).

#### 2.4.2. AMR Genes and Standard Curve Generation

The *bla*_CMY-2_, *bla*_CTX-M_, *qnrB19* and *mphA* resistance genes were quantified using quantitative real-time PCR in each pooled sample.

*E. coli* isolates previously sequenced (Table S2) that harbored the target genes were used as positive controls and templates for standard curve generation. The genomic DNA for the standard curve template was extracted using the DNeasy Blood and Tissue Kit (Qiagen, Valencia, CA, USA) and the QIAcube system (Qiagen, Valencia, CA, USA) following the manufacturer’s instructions. DNA quantities were measured using the Qubit dsDNA High Sensitivity Assay Kit in a Qubit 4 Fluorometer (Life Technologies, Carlsbad, CA, USA), following manufacturer instructions. Standard curve templates were serially 10-fold diluted, and each standard curve reaction contained 2 µL of diluted template DNA. The total genome size of the *E. coli* strain was 4,639,221 bp. Gene copy numbers per each gene were estimated using the following equation [30]:

(1) $$Copy\;number=\frac{Amount\;measured\;with\;Qubit\left(ng/µL\right)\times 6.022\times 10^{23}}{4,639,221\;bp\times 1\times 10^{9}\times 650}\times 2\;µL\;per\;reaction$$

#### 2.4.3. qPCR Reaction Setup and Gene Copy Number Analysis

DNA from the samples was distributed across the day, dairy farm, and treatment in 5 PCR plates per gene (Table S3). All procedures followed the Minimum Information for Publication of Quantitative Real-Time PCR Experiments (MIQE) guidelines [31].

Primers targeting the *bla*_CMY-2_, *bla*_CTX-M_, *qnrB*19, and 16S rRNA genes were previously published, and the thermal profile conditions are listed in Table 1. The primers and the probe targeting the *mphA* gene were designed using the PrimerQuest^TM^ Tool (Integrated DNA Technologies, Inc., Coralville, IA, USA) using sequences encoding *mphA* from the Comprehensive Antibiotic Resistance Database (https://card.mcmaster.ca/ontology/36455 accessed on 1 October 2021).

Reactions for the *bla*_CMY-2_, *bla*_CTX-M_, *qnrB*19, and 16S rRNA genes were composed of 10 μL of Brilliant III Ultra-Fast SBYR QPCR Master Mix with Low ROX (Agilent Technologies, Santa Clara, CA, USA), 3 μL of nuclease-free water (Qiagen, Valencia, CA, USA) and the corresponding forward and reverse primers with their concentrations for each gene as shown in Table 1. Reactions for the *mphA* gene were composed of 5 μL of water (Qiagen, Valencia, CA, USA), 10 μL of Brilliant III Ultra-Fast QPCR Master Mix with Low ROX (Agilent Technologies, Santa Clara, CA, USA), 2.5 μM of Forward and Reverse primers, and probe (FAM dye). The qPCR reactions were performed on the AriaMx Real-Time PCR system (Agilent Technologies, Santa Clara, CA, USA), and all community DNA was run and analyzed in duplicate. qPCR data were analyzed using the AriaMX ver 1.0 software (Agilent, Santa Clara, CA, USA) after each run, and the gene copy numbers per gram of wet feces were calculated from the gene quantity values derived from the Cq value in each sample, which were multiplied by 400 to account for the sample dilution and sample lost during the DNA extraction process (Table S4). Additionally, back-calculated gene copy numbers were log_10_ transformed and then standardized with the 16S rRNA gene by taking the ratio (i.e., log_10_ *bla*_CMY-2–_log_10_ 16S rRNA).

#### 2.4.4. Statistical Analysis

Descriptive analyses were performed using Stata version 17.1 (StataCorp LLC, College Station, TX, USA) for all standardized (and non-standardized) log_10_ transformed gene quantities. Linear regression was used for multivariate analysis of standardized (and non-standardized) log_10_ transformed gene copy numbers for all genes. Treatment, sample day, dairy farm, and replicate were considered fixed effects, along with the interaction term of a three-way full factorial model (no treatment, day 0, and dairy farm 1 was set as the baseline).

### 2.5. 16s rRNA Amplicon Gene Sequencing and Bioinformatic Analysis

#### 2.5.1. Total Community DNA Extraction and Library Preparation

The same DNA extracted from the 192 fecal pools used for qPCR were used for 16S rRNA metagenomic sequencing. The V3-V4 hypervariable region of the 16S rRNA gene (1550 bp) was amplified using the universal primers listed in Table 1. Sequencing libraries were prepared according to the Illumina 16S Metagenomic Sequencing Library Preparation Protocol [36] using the Illumina Nextera XT Library Preparation Kit and Indexes (Illumina, San Diego, CA, USA). DNA from the samples was distributed across the day, dairy farm, and treatment on two different sequencing runs (Table S5).

The final pooled library had a concentration of 4 nM, and 96 libraries were included in each run. Libraries were sequenced with the Illumina MiSeq platform using V3 kits with 300 bp paired end reads.

#### 2.5.2. Bioinformatic Analyses

A total of 16S rRNA raw reads were evaluated for quality using MultiQC [37] wrapper for FastQC [38]. Raw paired-end reads were analyzed using the QIIME2-2022.2 (Quantitative Insights Into Microbial Ecology 2022.2) pipeline by the Anaconda v3-5.0.0.1 module following the tutorial for QIIME 2 [39] on the High-Performance Research Computing System (HPRC) at Texas A&M University. FASTQ files were transformed into artifacts, and paired-end reads were quality-trimmed at a score of 16 (at 240 bp for forward and 248 bp reverse reads) to generate high-quality sequence variants. Moreover, samples with a depth of less than 10× were removed from the study for normalization purposes. Reads were classified using QIIME2 naïve Bayes classifier trained on the Silva database (v12_3) [40] to allocate the ASVs. To include only the domain bacteria in downstream analyses, we filtered out the Archaea, Eukaryota, Mitochondria, and Chloroplast taxa. Additionally, to evaluate the differences in bacterial taxonomic composition, we calculated the Shannon index to estimate the alpha diversity between treatment groups, farms, and days, using a linear regression model to account for treatment, sample day, farm, and plate as fixed factors, along with the three-way full factorial model (no treatment, day 0, and dairy farm 1 set as baseline). Principal component analysis (PCoA) using weighted and unweighted UniFrac indices was computed for beta diversity. The permutational analysis of variance (PERMANOVA) test was used to determine statistical differences between treatment groups using the Vegan package in R (version 2.6-4). A rarefaction depth of 11783 sequences per sample was used for alpha- and beta-diversity analyses. Finally, to identify significant differences in the relative abundance of taxa between treatment groups, the Linear Discriminant Analysis Effect Size (LEfSe) analysis with the Kruskal–Wallis test was used (https://huttenhower.sph.harvard.edu/galaxy/ accessed on 20 July 2023) using a of *p*-value of 0.05 and LDA (Linear Discriminatory Analysis) of 3.0 [41].

## 3. Results

### 3.1. Quantification of bla_CMY-2_, bla_CTX-M_, mphA and qnrB19

#### 3.1.1. Descriptive Statistics

In total, there were 728 fecal samples from 124 matched pairs of cows over 16 days, and the samples were pooled (*n* = 192) in groups of 4 by day and treatment (Table S1). To determine the effects of 2-CCFA on the resistance genes *bla_CMY-2_*, *bla_CTX-M_*, *mphA*, *and qnrB19*, we quantified and standardized the gene copies per gram feces from community DNA extracted from the 192 fecal pools. Day 6 represented the maximum expected effect of 2-CCFA treatment, and day 16 represented the first eligible day of slaughter following treatment compared to the baseline levels on day 0 before treatment.

Among the 192 fecal pools tested by qPCR in duplicate (i.e., 384 reactions), *mphA* represented the highest mean standardized (to 16S rRNA copies) log_10_ gene copies/gram feces (−5.04) with 346 detections, followed by *bla*_CMY-2_ (−5.73) with 348 detections, *qnrB*19 (−5.91) with 383 detections, and, finally, *bla_CTX-M_* (−6.29) with 352 detections (Table 2). The 16S rRNA was quantifiable for all samples and was the basis for standardization.

There was a trend of mean increase over time for the *bla*_CMY-2_ gene from day 0 to day 16. Furthermore, samples from dairy farm 3 exhibited a higher mean and median of *bla*_CMY-2_ log_10_ gene copies/gram feces; however, the mean values for the other genes were lower on this farm. Descriptive statistics on the distribution of non-standardized gene detections across treatment and sampling days are presented in Table S6.

#### 3.1.2. Detection of *bla_CMY-2_*, *bla_CTX-M_*, *mphA* and *qnrB19* Genes

**Figure 2:**
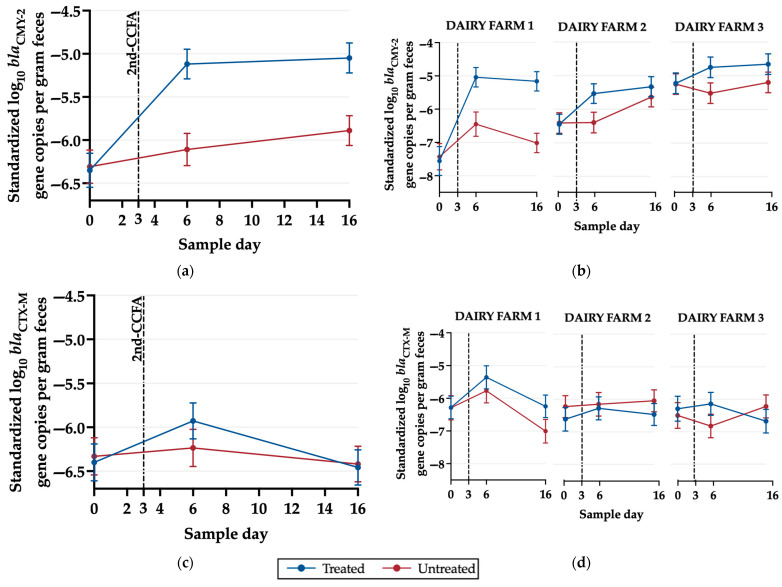
Marginal means graphs of the linear regression model of standardized (log_10_ target resistance gene–log_10_ 16S rRNA) log_10_ genes copies per gram of feces based on (**a**) *bla*_CMY-2_, (**b**) *bla*_CMY-2_ by farm, (**c**) *bla*_CTX-M_, and (**d**) *bla*_CTX-M_ by farm. Data were analyzed using Stata/BE version 17.0 and graphed using GraphPad Prism version 10.0.2.

A linear regression was used to evaluate non-standardized (Figure S1) and standardized gene copy numbers on a log_10_ scale across sampling days. There was a significant increase (*p* ≤ 0.05) in *bla*_CMY-2_ gene copies for treated animals on days 6 and 16 compared with day 0 (Figure 2a). The *bla*_CMY-2_ levels significantly differed between treated and untreated animals on day 6 on all dairy farms. Also, *bla*_CMY-2_ log_10_ gene copies significantly differed between treated and untreated on day 16 on dairy farm 1 (Figure 2b). In contrast, log_10_ *bla_CTX-M_* gene copies only significantly differed between treated and untreated animals on dairy farm 1 on day 16 (Figure 2d), and log_10_ *mphA* and *qnrB19* gene copies were not significantly different between treatments, farms, or days (Figure S2).

### 3.2. Bacterial 16S Metagenomics

#### 3.2.1. Descriptive Statistics

A pipeline for metagenomics analyses was used to understand the day-by-day differences in the fecal microbiota between dairy cattle with metritis treated with 2-CCFA and an untreated group without metritis on three farms, using the V3-V4 hyper-variable region of the 16S rRNA gene. The observed quality distribution from MultiQC showed that most reads had a Q-score of 25 or higher, indicating no quality problems (Figure S3). A total of 27,473,717 reads (an average of 143,092 ranging from 9345 to 381,656 reads per sample) were obtained. After quality control (trimming and filtering) of reads, 188 out of 192 samples were retained, with approximately 9,927,829 raw reads remaining for subsequent analysis. Within the 4 samples lost due to sampling depth, 1 belonged to dairy farm 1 (treated cow fecal pool on day 16), and 3 belonged to dairy farm 2 (2 treated cow pools on day 0 and 1 untreated cow pool on day 16).

Rarefaction curves reached a plateau at 250 observed features, indicating that the sequencing effort was suitable to describe most of the taxa present (Figure S4).

#### 3.2.2. Microbial Compositional Analyses

**Figure 3:**
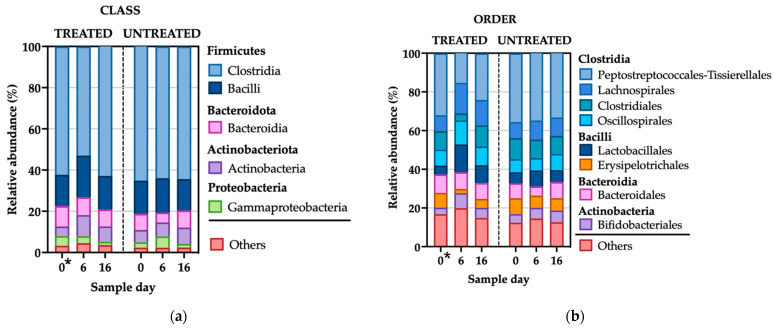
Fecal microbiome relative abundance by day and treatment (2-CCFA). Comparisons of relative abundance at the level of (**a**) class and (**b**) order. * On day 0, the only difference between the treatment groups was the presence of metritis in the treated group. The graph was generated using GraphPad Prism version 10.0.2.

After quality trimming, 9,927,829 bacterial ASVs were found in the 188 fecal pools. There were 57 different bacterial classes in total identified in both treated and untreated animals, with Clostridia (61.90%) being the most abundant, followed by Bacilli (16.66%), Bacteroidia (7.90%), Actinobacteria (7.18%), and Gammaproteobacteria (3.27%). The remaining bacterial classes showed a relative abundance of ≤1% each (Figure 3a). There were 120 different bacterial orders in total identified in both treated and untreated animals. Peptostreptococcales-Tissierellales (29.11%) was the most abundant, followed by Lachnospirales (10.88%), Clostridiales (8.95%), Oscillospirales (8.65%), and Lactobacillales (8.12%), Bacteroidales (7.83%), Erysipelotrichales (5.86%), Bifidobacteriales (5.44%), Pseudomonadales (2.92%), Bacillales (2.24%), Christensenellales (2.16%), Micrococcales (1.61%), Clostridia_UCG-014 (1.18%). The remaining bacterial orders had a relative abundance of <1% (Figure 3b). Additionally, the comparisons of fecal microbiome relative abundance by day and treatment at the bacterial phylum and family levels are presented in Figures S5 and S6, which display the taxonomic levels by dairy farm.

The treated animals showed higher relative abundance for the most abundant taxa at the levels of class, order, and family on days 6 and 16 compared with the untreated group. The most dramatic change was observed where the Clostridia class decreased in relative abundance while Bacilli and Actinobacteria classes increased, specifically in the 2-CCFA treated group on day 6 (Figure 3a). The relative abundance of the taxa in the untreated group remained similar on days 0, 6, and 16.

**Figure 4:**
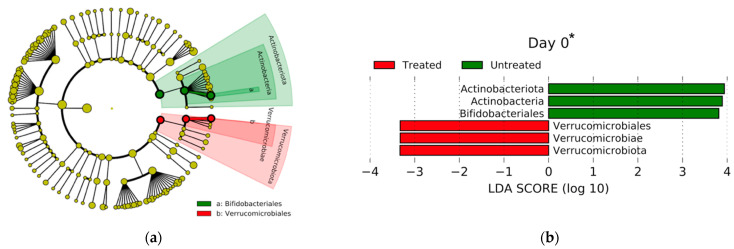

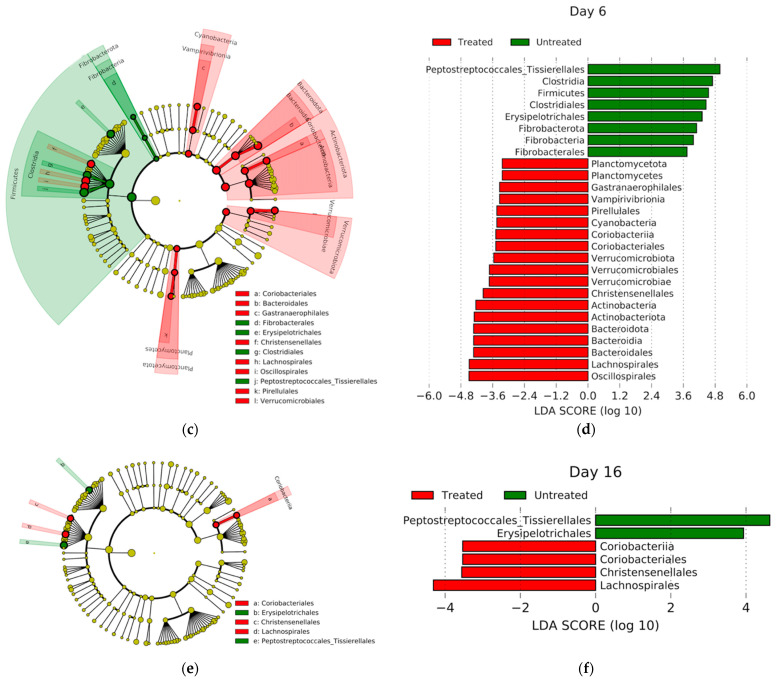
Linear Discriminant Analysis Effect Size (LEfSe) and taxonomic cladogram results of the significantly differentially abundant taxa in treated and untreated groups on day 0 (**a**,**b**), day 6 (**c**,**d**), and day 16 (**e**,**f**). Orders more abundant in the treated group are presented in red (indicated with a negative Linear Discriminant Analysis (LDA) score, whereas orders more abundant in the untreated group are presented in green (indicated with a positive LDA score). In the cladogram, the size of each dot is proportional to the effect size. * On day 0, the only difference between the treated groups is the presence of the animal disease metritis.

LEfSe identified the taxa that were significantly enriched by comparing treated and untreated animals at the order level across all farms (Figure 4). On day 0, the relative abundances of Actinobacteriota were significantly enriched in the untreated group, compared to the relative abundances of Verrucomicrobiota, which were significantly enriched in the treated group. On day 6, the relative abundances of Peptostreptococcales-Tissierellales were significantly enriched in the untreated group, compared to the relative abundances of Lachnospirales and Oscillospirales, which were significantly enriched in the treated group. Lastly, on day 16, the relative abundances of Peptostreptococcales-Tissierellales and Erysipelotrichales were significantly enriched in the untreated group, compared to the relative abundances of Lachnospirales, which were significantly enriched in the treated group.

#### 3.2.3. Alpha Diversity

**Figure 5:**
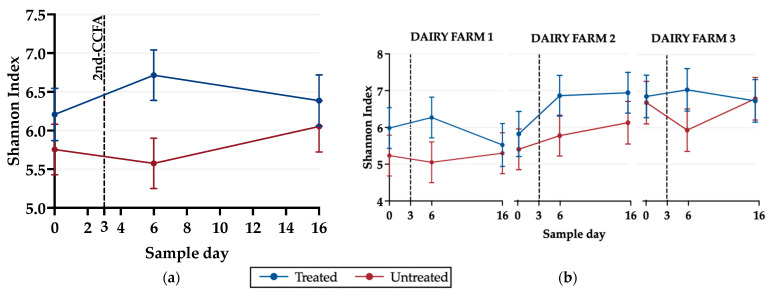
Marginal means graphs from a linear regression model of the Shannon alpha diversity index in treated and untreated groups on (**a**) days 0, 6, and 16 and (**b**) by farm. Data were analyzed using Stata/BE version 17.0 and graphed using GraphPad Prism version 10.0.2.

Fecal samples from treated animals had significantly greater (*p* ≤ 0.05) richness and evenness than the untreated group on day 6 as measured through the Shannon Index (Figure 5a). Additionally, the Shannon Index only significantly differed (*p* ≤ 0.05) between treated and untreated animals on dairy farm 1 on day 6. In contrast, there were no significant differences (*p* > 0.05) between treatments and days on dairy farms 2 and 3 (Figure 5b).

#### 3.2.4. Beta Diversity (Community Comparison)

**Figure 6:**
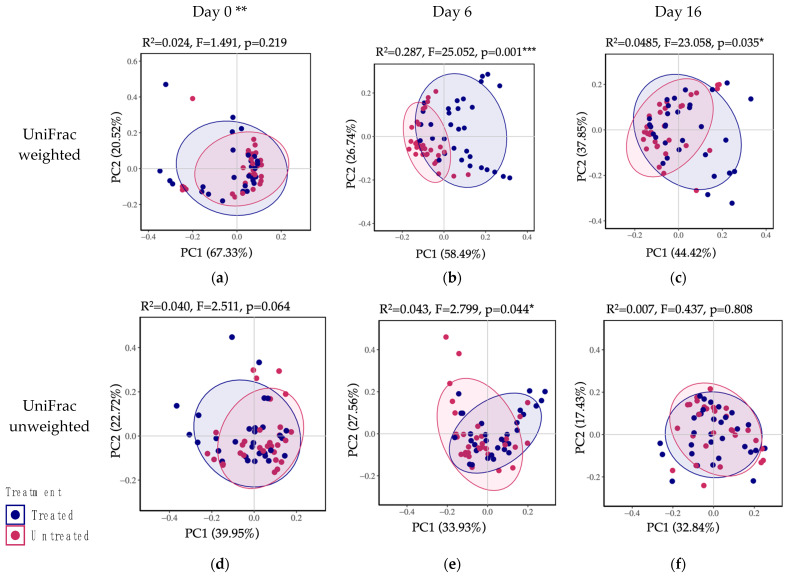
Fecal microbial beta-diversity represented in Principal Component Ordinal Analyses (PCoA) clustered by day and treatment. UniFrac distances between bacterial community composition based on (**a**–**c**) weighted (quantitative) and (**d**–**f**) unweighted (qualitative) on days 0, 6, and 16, respectively. Figures were generated using the Vegan package in R Studio. ** On day 0, the only difference between the treatment groups is the presence of the disease metritis in the (not-yet) treated group. *** Significant at *p* < 0.001, * Significant at *p* < 0.01.

According to the principal coordinate analysis (PCoA) plots of weighted and unweighted UniFrac performed using the Bray-Curtis dissimilarity index (Figure 6), the treated and untreated groups presented similar bacteria communities on day 0 (*p* = 0.219 on Figure 6a; *p* = 0.064 on Figure 6d); however, on day 6, the bacterial communities were significantly different between treatment groups (*p* = 0.001 on Figure 6b; *p* = 0.044 on Figure 6e). On day 16, there were significant differences between treatments in the weighted UniFrac (*p* = 0.035 in Figure 6c), but no significant differences were found on day 16 on unweighted UniFrac (*p* = 0.808 in Figure 6f). Interestingly, PCoA results by dairy farm (Figure S7) suggested that only dairy farm 3 did not present significant differences between days (weighted, PERMANOVA *p* = 0.145) and treatments (weighted PERMANOVA *p* = 0.217). Additionally, fecal microbial beta-diversity represented in PCoA by treatment, day, and dairy are presented in Figure S8.

## 4. Discussion

This study was designed to assess the impact of a two-dose administration of ceftiofur crystalline-free acid (2-CCFA) on the fecal microbiota and the standardized quantities of third-generation cephalosporin, fluoroquinolone, and macrolide resistance genes in Holstein-Friesian dairy cows across three large dairy farms in the southwestern United States. To achieve this, our culture-independent methodology involved 16S metagenomics to analyze the microbial communities and qPCR to quantify the levels of the *bla*_CMY-2_, *bla*_CTX-M_, *qnrB*19, and *mphA* genes within the feces, standardized to 16S rRNA gene copies representing the bacterial populations. Overall, there was a significant increase in microbial diversity by day 6 in the 2-CCFA-treated group based on alpha and beta diversity. Additionally, the *bla*_CMY-2_ gene copies/gram feces increased significantly in the treated group compared to the untreated group on day 6 and remained significantly elevated on the first day of slaughter eligibility on day 16.

### 4.1. Two-Dose CCFA Favors the Selection of the bla_CMY-2_ Gene

One of the major findings in this study was the significant impact of 2-CCFA on the quantity of the log_10_ *bla*_CMY-2_ gene in the treated animals on day 6 and day 16 in the linear regression model collapsed across the farms (Figure 2a). In contrast, no significant differences were observed for treatment, day, or farm for the *bla*_CTX-M_, *mphA*, or *qnrB19* genes. Similar to our findings, Kanwar et al., (2014) found a significant increase in *bla*_CMY-2_ and *bla*_CTX-M_ gene copies per gram of feces in beef cattle treated with a single-dose of CCFA. However, in contrast to the current study, Kanwar et al. (2014) observed a one-fold reduction in starting levels of *bla*_CMY-2_ gene copy numbers, suggesting that the resistance levels may have increased over time (from 2009 through 2018). Another result similar to the current study, Alali et al. (2009) observed that using CCFA in feedlot cattle significantly increased the *bla*_CMY-2_ gene numbers. It is important to note that these studies vary between production types (beef versus dairy) and antibiotic treatment regimen. Kanwar et al. (2014) used a single dose of 6.6 mg/kg, while Alali et al. (2009) used the same dosage but with a three-dose administration on days 0, 6, and 13 [42,43]. The latter protocol would be in violation of federal statutes passed in 2012, when such extra-label uses were banned in the United States.

In the current study, the notable increase in the *bla*_CMY-2_ gene in the treated group on the first eligible slaughter day (day 16; 13 days following the second dose of CCFA) relative to baseline levels on day 0 indicates the potential risk for this gene to transfer from livestock to humans through the food chain, potentially leading to wider dissemination [44]. This is important because CCFA is a third-generation cephalosporin, and this class of antibiotics is considered to be a highest priority/critically important antimicrobial in human medicine by the WHO [27]. Notably, a similar molecule, ceftriaxone, is a third-generation cephalosporin used in human medicine to treat severe cases of salmonellosis, and *Salmonella* is a significant foodborne pathogen commonly found in cattle feces [45,46].

Although there was no observed significant effect of 2-CCFA treatment on the quantities of the *bla*_CTX-M_, *mphA*, and *qnrB19* genes (Figure S2), and these genes did not present any differences by day, treatment, or farm, the co-selection of *qnrB19* and *mphA* through 3GC genes would likely be influenced mainly through their association with *bla*_CTX-M_ rather than *bla*_CMY-2_ [26]. Further exploration of the plasmids may prove how 2-CCFA selection pressures affect the different genes. Previous studies have identified IncN plasmids carrying *bla*_CTX-M_ and *qnrB19* genes [47,48]. Additionally, in a study of fecal samples from dairy calves, associations were found between *bla*_CTX-M-1_, *mphA,* and *qnrB* [34], and, interestingly, it was found that *mphA* and *qnrB* were associated with different variants of *bla*_CTX-M_. Therefore, studying the association between the gene and the mobile genetic elements may provide better insight into this question. Moreover, while *bla*_CTX-M_ was first described in 1989 in humans [49] and first reported in livestock animals in 2010 by Wittum et al. from the feces of sick and healthy cattle in Ohio [50], *bla*_CMY-2_ was first described in 1990 [51] and first reported in livestock animals in 2000 by Fey et al., from cattle in Nebraska [52]. Therefore, the *bla*_CMY-2_ gene has been present longer in USA agricultural animals than *bla*_CTX-M_, likely leading to a greater potential for selection of the cephamycinase gene.

### 4.2. The Feces from Treated Animals Presented a Significantly Higher Microbial Diversity

Although this study is the first pair-matched and controlled field trial to describe the impact of 2-CCFA on the fecal microbiome of dairy cattle diagnosed with metritis, the 16S rRNA sequencing data revealed that Firmicutes and Bacteroidetes phyla constitute around 90% of the cattle fecal microbiome (Figure S5); furthermore, this finding is consistent with previous studies on the microbiome of dairy cattle feces [53]. The overall differences in the bacterial composition between the cattle treated with 2-CCFA and the untreated cattle on day 6 are likely due to the reduction in the predominant Clostridia class and the increase in the Bacilli and Actinobacteria classes (Figure 3b), which resulted in a measure of overall greater alpha diversity (Figure 5a), which should not necessarily be interpreted as being beneficial to the host [54]. These perhaps unexpected results may indicate that 2-CCFA, due to its bactericidal properties, led to a reduction in certain dominant classes of bacteria, including those that may be beneficial to the microbiome, and led to an increase in other bacterial taxa, thus creating a potential dysbiosis. Previous research [55] has shown that most species of the *Bacillus* genera degrade ceftiofur, which could contribute to the observed increase in these genera.

We did not study the impact of 2-CCFA on the bacterial populations present in the microbiome after day 16, and we were only able to observe the effect of 2-CCFA on the abundance of the fecal bacterial communities on day 16 compared with days 0 and 6. As presented in the figures of weighted UniFrac (which account for bacterial presence/absence and their abundance) (Figure 6a–c), the bacterial communities remained significantly different on days 6 and 16 compared with day 0. However, when comparing the unweighted UniFrac (which accounts only for bacterial presence), the bacterial communities did not remain significantly different on day 16 (Figure 6d–f), suggesting the bacteria present in the microbiome recovered somewhat to untreated community profile levels. However, the abundance of the taxa differed between the treated group and the untreated group.

### 4.3. Limitations

The limitations of this study include potential confounders that may have influenced our results, such as age, season, and even the fecal pools. Another limitation would be the generalizability of the results due to the small number of dairy farms enrolled in our study and differences in farm-level practices. Figure 2 shows that there are variations in *bla*_CMY-2_ and *bla*_CTX-M_ log_10_ gene copies among different dairy farms, which suggests that farm-level practices, such as the frequency of CCFA historical usage, may have an impact on the study; however, it is challenging to determine the pre-existing differences on day 0. Furthermore, the presence of metritis in the treatment group could impact the observed differences in bacterial taxa because the treated group was diagnosed with metritis before receiving 2-CCFA, and the untreated group was selected for their lack of metritis diagnosis. It is important to acknowledge that the absence of a group without metritis administered 2-CCFA may introduce bias into our results. Nevertheless, ceftiofur in healthy food-producing animals is strictly prohibited by U.S. FDA regulation (21 CFR Part 530). Leaving untreated cows diagnosed with metritis was not deemed ethical or in the interests of animal welfare; furthermore, the farm owners would not permit it. Therefore, the treatment of animals was limited to those diagnosed with metritis, and no negative control group was included.

Additionally, the information obtained from 16S rRNA amplicon sequencing offers a limited view of bacterial composition because of the restricted taxonomic resolution, limited functional analysis, and lower sensitivity compared to 16S shotgun metagenomic data, which would also capture the relative quantities of target resistance genes [56]. These limitations underline the importance of further exploring the genes present in the samples and how they are associated. Incorporating 16S shotgun metagenomics could enrich this study by allowing for a more in-depth analysis of the functional metagenomic composition and antimicrobial resistance between treatment groups, as presented in previous studies [57,58] that evaluated the prevalence of antimicrobial resistance in the fecal microbiome of dairy cattle.

## 5. Conclusions

Our study provides insight into the complex relationships between antibiotic use, gene propagation and decline, and microbial diversity. We found that administering 2-CCFA creates a selection pressure that expands the cephamycinase gene *bla*_CMY-2_ through the first eligible day of slaughter. Though not observed here, there may be potential for co-selection between *bla*_CTX-M_, *qnrB*, and *mphA* when *bla*_CTX-M_ is present. Furthermore, 2-CCFA in dairy cattle diagnosed with metritis likely resulted in a dysbiosis of the fecal microbiome caused by a significant increase in diversity after treatment. This was due to the decrease in the dominant healthy gut taxa, Clostridia, and the concomitant increase in Bacilli and Actinobacteria. In conclusion, administering 2-CCFA to dairy cows with metritis elevates cephamycinase gene quantities among all fecal bacteria while paradoxically increasing microbial diversity. In the absence of the curing effect for metritis, the latter effect should not necessarily be interpreted as being in the best interests of the host.

## Figures and Tables

**Table 1:** Primers and probes to quantify AMR genes used for qPCR.

Target Gene	Primers and Probes Sequences	Amount (μM)	Annealing Tm (°C)	Product Size (bp)	References
*bla*_CMY-2_	F 5′-TTCTCCGGGACAACTTGACG-3′ R 5′-GCATCTCCCAGCCTAATCCC-3′	1	60	210	[32]
*bla*_CTX-M_	F 5′-CTATGGCACCACCAACGATA-3′ R 5′-ACGGCTTTCTGCCTTAGGTT-3′	5	60	103	[33]
*mphA*	F-5′GCG AAG GTC GAA CCA GAG-3′ R-5′GAG TCT TCG AGC ATG GGA TAG-3′ P-5′56-FAM/CGA TTC TTG/ZEN/AGCATT GCC AGC ACC/3IABkFQ/-3′	2.5	60	119	This study
*qnrB19*	F 5′-CAC ATT ATC GCG TGA CCA ATT-3′ R 5′-GAT GCC TGG TAG CTG TCT AAC-3′	1	58	91	Modified from [34]
16S rRNA	F 5′-CCTACGGGNGGCWGCAG-3′ R 5′-GACTACHVGGGTATCTA ATCC-3′	3.5	58	464	[35]

**Table 2:** Descriptive statistics on the distribution of overall run summary gene detections within treatment, sampling day, and dairy farm.

Standardized Gene Target (Log_10_)	Descriptive Statistics	Overall Detections * (N)	Treatment		Sample day			Dairy Farm		
			Treated	Untreated	0	6	16	1	2	3
*bla*_CMY−2_	Observations	348	170	178	103	118	127	102	126	120
	Mean	−5.73	−5.40	−6.08	−6.19	−5.56	−5.52	−6.21	−5.95	−5.10
	Median	−5.65	−5.36	−6.13	−6.30	−5.61	−5.49	−6.29	−6.04	−5.13
	95% CI	−5.84/−5.62	−5.56/−5.25	−6.22/−5.93	−6.41/−5.97	−5.72/−5.41	−5.7/−5.34	−6.45/−5.98	−6.08/−5.81	−5.24/−4.95
	Standard error	0.06	0.08	0.07	0.11	0.08	0.09	0.12	0.07	0.07
*bla*_CTX−M_	Observations	352	179	173	113	116	123	117	124	111
	Mean	−6.29	−6.26	−6.33	−6.36	−6.08	−6.43	−6.16	−6.29	−6.44
	Median	−6.38	−6.37	−6.42	−6.45	−6.14	−6.57	−6.19	−6.47	−6.48
	95% CI	−6.38/−6.2	−6.39/−6.12	−6.45/−6.21	−6.51/−6.2	−6.25/−5.92	−6.57/−6.28	−6.33/−5.98	−6.42/−6.16	−6.6/−6.28
	Standard error	0.05	0.07	0.06	0.08	0.08	0.07	0.09	0.07	0.08
*mphA*	Observations	346	172	174	114	116	116	124	123	99
	Mean	−5.04	−5.00	−5.07	−5.11	−4.93	−5.08	−5.01	−4.85	−5.30
	Median	−5.15	−5.09	−5.18	−5.16	−5.03	−5.26	−5.11	−4.91	−5.48
	95% CI	−6.01/−5.82	−5.12/−4.88	−5.2/−4.95	−5.24/−4.98	−5.07/−4.8	−5.26/−4.89	−5.17/−4.85	−4.99/−4.71	−5.44/−5.17
	Standard error	0.05	0.06	0.06	0.07	0.07	0.09	0.08	0.07	0.07
*qnrB19*	Observations	383	191	192	127	128	128	132	132	119
	Mean	−5.91	−5.86	−5.97	−5.90	−5.92	−5.92	−5.61	−5.69	−6.50
	Median	−5.96	−5.98	−5.95	−5.95	−5.92	−6.14	−5.79	−5.76	−6.59
	95% CI	−6.01/−5.82	−5.99/−5.73	−6.1/−5.84	−6.05/−5.75	−6.07/−5.77	−6.1/−5.74	−5.78/−5.45	−5.82/−5.56	−6.64/−6.35
	Standard error	0.05	0.07	0.07	0.08	0.08	0.09	0.08	0.07	0.07

* N total = 192 pools in replicate = 384 runs per gene target.

## Data Availability

The raw sequencing data is available in the NCBI under the project number PRJNA625290.

## Supplementary Materials

The following supporting information can be downloaded at: https://www.mdpi.com/article/10.3390/microorganisms11112728/s1, Table S1. 744 fecal samples across days 0, 6, and 16, pooled in four groups by dairy farm, day, and treatment; Table S2. *E. coli* isolates previously sequenced that harbored the target genes used as positive controls and templates for qPCR standard curve generation; Table S3. DNA extracted from the fecal samples distributed across the day, dairy, and treatment in 5 PCR plates per gene; Table S4. Fecal sample dilution and sample loss during the DNA extraction process; Table S5. DNA distribution from the fecal samples across the day, dairy, and treatment on two different sequencing runs; Table S6. Descriptive statistics on the distribution of non-standardized gene detections across treatment and sampling days; Figure S1. Marginal means graphs showing the linear regression model of non-standardized log_10_ genes copies per gram of feces based on (a) *bla*_CMY-2_, (b) *bla*_CTX-M_, (c) *mphA*, and (d) *qnrB19*; Figure S2. Marginal means graphs showing the linear effect model of 16S rRNA-standardized log_10_ genes copies per gram of feces based on (a) *mphA* and (b) *mphA* by the farm. (a) *qnrB19*, and (b) *qnrB19* by farm; Figure S3. Per sequences quality scores; Figure S4. Rarefaction curves displayed by treatment; Figure S5. Fecal microbiome relative abundance phylogenetic profile by day and treatment. Comparisons of relative abundance within the bacteria domain at the level of bacterial (A) phylum and (B) family; Figure S6. Fecal microbiome relative abundance phylogenetic profile by dairy farm, day, and treatment. Comparisons of relative abundance at the level of bacterial (A) phylum, (B) class, and (C) order; Figure S7. Fecal microbial beta-diversity represented in Principal Component Ordinal Analyses (PCoA) by dairy farm and clustered by day and treatment. UniFrac distances between bacterial community composition based on (top three) weighted (quantitative) and (bottom three) unweighted (qualitative); Figure S8. Fecal microbial beta-diversity represented in Principal Component Ordinal Analyses (PCoA) by dairy, day, and treatment. UniFrac distances between bacterial community composition based on (top three) weighted (quantitative) and (bottom three) unweighted (qualitative).

## References

[1] Fleming A. (1929). Classics in infectious diseases: On the antibacterial action of cultures of a penicillium, with special reference to their use in the isolation of B. influenzae by Alexander Fleming. Br. J. Exp. Pathol..

[2] Prestinaci F., Pezzotti P., Pantosti A. (2015). Antimicrobial resistance: A global multifaceted phenomenon. Pathog. Glob. Health.

[3] CDC (2019). Antibiotic Resistance Threats in the United States.

[4] O’Neil J. (2016). Tackling Drug-Resistant Infections Globally: Final Report and Recommendations.

[5] Castanheira M., Simner P.J., Bradford P.A. (2021). Extended-spectrum beta-lactamases: An update on their characteristics, epidemiology and detection. JAC Antimicrob. Resist..

[6] Davies J., Davies D. (2010). Origins and evolution of antibiotic resistance. Microbiol. Mol. Biol. Rev..

[7] Antimicrobial Resistance C. (2022). Global burden of bacterial antimicrobial resistance in 2019: A systematic analysis. Lancet.

[8] Basavaraju M., Gunashree B.S. (2022). Escherichia coli: An Overview of Main Characteristics.

[9] Poirel L., Madec J.Y., Lupo A., Schink A.K., Kieffer N., Nordmann P., Schwarz S. (2018). Antimicrobial Resistance in *Escherichia coli*. Microbiol. Spectr..

[10] Braz V.S., Melchior K., Moreira C.G. (2020). *Escherichia coli* as a Multifaceted Pathogenic and Versatile Bacterium. Front. Cell Infect. Microbiol..

[11] Huddleston J.R. (2014). Horizontal gene transfer in the human gastrointestinal tract: Potential spread of antibiotic resistance genes. Infect. Drug Resist..

[12] Rozwandowicz M., Brouwer M.S.M., Fischer J., Wagenaar J.A., Gonzalez-Zorn B., Guerra B., Mevius D.J., Hordijk J. (2018). Plasmids carrying antimicrobial resistance genes in Enterobacteriaceae. J. Antimicrob. Chemother..

[13] Jacoby G.A. (2009). AmpC beta-lactamases. Clin. Microbiol. Rev..

[14] Xiang Y., Wu F., Chai Y., Xu X., Yang L., Tian S., Zhang H., Li Y., Yang C., Liu H. (2020). A new plasmid carrying mphA causes prevalence of azithromycin resistance in enterotoxigenic *Escherichia coli* serogroup O6. BMC Microbiol..

[15] Stanton I.C., Murray A.K., Zhang L., Snape J., Gaze W.H. (2020). Evolution of antibiotic resistance at low antibiotic concentrations including selection below the minimal selective concentration. Commun. Biol..

[16] Phuc Nguyen M.C., Woerther P.L., Bouvet M., Andremont A., Leclercq R., Canu A. (2009). *Escherichia coli* as reservoir for macrolide resistance genes. Emerg. Infect. Dis..

[17] Worthington R.J., Melander C. (2013). Overcoming resistance to beta-lactam antibiotics. J. Org. Chem..

[18] Bush K., Bradford P.A. (2016). beta-Lactams and beta-Lactamase Inhibitors: An Overview. Cold Spring Harb. Perspect. Med..

[19] Ramirez J., Guarner F., Bustos Fernandez L., Maruy A., Sdepanian V.L., Cohen H. (2020). Antibiotics as Major Disruptors of Gut Microbiota. Front. Cell Infect. Microbiol..

[20] Merenda V.R., Lezier D., Odetti A., Figueiredo C.C., Risco C.A., Bisinotto R.S., Chebel R.C. (2021). Effects of metritis treatment strategies on health, behavior, reproductive, and productive responses of Holstein cows. J. Dairy. Sci..

[21] Perez-Baez J., Silva T.V., Risco C.A., Chebel R.C., Cunha F., De Vries A., Santos J.E.P., Lima F.S., Pinedo P., Schuenemann G.M. (2021). The economic cost of metritis in dairy herds. J. Dairy. Sci..

[22] Taylor E.A., Jordan E.R., Garcia J.A., Hagevoort G.R., Norman K.N., Lawhon S.D., Pineiro J.M., Scott H.M. (2019). Effects of two-dose ceftiofur treatment for metritis on the temporal dynamics of antimicrobial resistance among fecal Escherichia coli in Holstein-Friesian dairy cows. PLoS ONE.

[23] Samtiya M., Matthews K.R., Dhewa T., Puniya A.K. (2022). Antimicrobial Resistance in the Food Chain: Trends, Mechanisms, Pathways, and Possible Regulation Strategies. Foods.

[24] (2019). Draxxin Cattle Prescribing Information. https://www.zoetisus.com/content/pages/products/cattle/Draxxin-resources/Assets/Draxxin-Cattle-Prescribing-Information.pdf.

[25] (2021). Enroflox 100 Package Insert. https://www.norbrook.com/media/2873/enroflox-100-package-insert-6405101670i07.pdf.

[26] Taylor E.A., Ossa-Trujillo C., Vinasco J., Jordan E.R., Garcia Buitrago J.A., Hagevoort R., Norman K.N., Lawhon S.D., Pineiro J.M., Levent G. (2021). Use of critically important antimicrobial classes early in life may adversely impact bacterial resistance profiles during adult years: Potential co-selection for plasmid-borne fluoroquinolone and macrolide resistance via extended-spectrum beta-lactam use in dairy cattle. Lett. Appl. Microbiol..

[27] WHO (2019). Critically Important Antimicrobials for Human Medicine.

[28] Scott H.M., Acuff G., Bergeron G., Bourassa M.W., Gill J., Graham D.W., Kahn L.H., Morley P.S., Salois M.J., Simjee S. (2019). Critically important antibiotics: Criteria and approaches for measuring and reducing their use in food animal agriculture. Ann. N. Y. Acad. Sci..

[29] Code of Federal Regulations (2012). Chapter I. Food and Drug Administration; Subchapter E. Animal drugs, feeds, and related products; Part 530. Extralabel drug use in animals. Food and Drugs.

[30] Brankatschk R., Bodenhausen N., Zeyer J., Burgmann H. (2012). Simple absolute quantification method correcting for quantitative PCR efficiency variations for microbial community samples. Appl. Environ. Microbiol..

[31] Bustin S.A., Benes V., Garson J.A., Hellemans J., Huggett J., Kubista M., Mueller R., Nolan T., Pfaffl M.W., Shipley G.L. (2009). The MIQE guidelines: Minimum information for publication of quantitative real-time PCR experiments. Clin. Chem..

[32] Vikram A., Rovira P., Agga G.E., Arthur T.M., Bosilevac J.M., Wheeler T.L., Morley P.S., Belk K.E., Schmidt J.W. (2017). Impact of “Raised without Antibiotics” Beef Cattle Production Practices on Occurrences of Antimicrobial Resistance. Appl. Environ. Microbiol..

[33] Marti E., Jofre J., Balcazar J.L. (2013). Prevalence of antibiotic resistance genes and bacterial community composition in a river influenced by a wastewater treatment plant. PLoS ONE.

[34] Carey A.M., Capik S.F., Giebel S., Nickodem C., Pineiro J.M., Scott H.M., Vinasco J., Norman K.N. (2022). Prevalence and Profiles of Antibiotic Resistance Genes mph(A) and qnrB in Extended-Spectrum Beta-Lactamase (ESBL)-Producing Escherichia coli Isolated from Dairy Calf Feces. Microorganisms.

[35] Klindworth A., Pruesse E., Schweer T., Peplies J., Quast C., Horn M., Glockner F.O. (2013). Evaluation of general 16S ribosomal RNA gene PCR primers for classical and next-generation sequencing-based diversity studies. Nucleic Acids Res..

[36] Illumina (2013). Illumina 16S Metagenomic Sequencing Library Preparation (Illumina Technical Note 15044223). http://support.illumina.com/documents/documentation/chemistry_documentation/16s/16s-metagenomic-library-prep-guide-15044223-b.pdf.

[37] Ewels P., Magnusson M., Lundin S., Kaller M. (2016). MultiQC: Summarize analysis results for multiple tools and samples in a single report. Bioinformatics.

[38] Andrews S. (2010). FastQC: A Quality Control Tool for High Throughput Sequence Data. http://www.bioinformatics.babraham.ac.uk/projects/fastqc.

[39] Estaki M., Jiang L., Bokulich N.A., McDonald D., Gonzalez A., Kosciolek T., Martino C., Zhu Q., Birmingham A., Vazquez-Baeza Y. (2020). QIIME 2 Enables Comprehensive End-to-End Analysis of Diverse Microbiome Data and Comparative Studies with Publicly Available Data. Curr. Protoc. Bioinformatics.

[40] Bokulich N.A., Kaehler B.D., Rideout J.R., Dillon M., Bolyen E., Knight R., Huttley G.A., Gregory Caporaso J. (2018). Optimizing taxonomic classification of marker-gene amplicon sequences with QIIME 2’s q2-feature-classifier plugin. Microbiome.

[41] Segata N., Izard J., Waldron L., Gevers D., Miropolsky L., Garrett W.S., Huttenhower C. (2011). Metagenomic biomarker discovery and explanation. Genome Biol..

[42] Kanwar N., Scott H.M., Norby B., Loneragan G.H., Vinasco J., Cottell J.L., Chalmers G., Chengappa M.M., Bai J., Boerlin P. (2014). Impact of treatment strategies on cephalosporin and tetracycline resistance gene quantities in the bovine fecal metagenome. Sci. Rep..

[43] Alali W.Q., Scott H.M., Norby B., Gebreyes W., Loneragan G.H. (2009). Quantification of the bla(CMY-2) in feces from beef feedlot cattle administered three different doses of ceftiofur in a longitudinal controlled field trial. Foodborne Pathog. Dis..

[44] Bennani H., Mateus A., Mays N., Eastmure E., Stark K.D.C., Hasler B. (2020). Overview of Evidence of Antimicrobial Use and Antimicrobial Resistance in the Food Chain. Antibiotics.

[45] Castanheira S., Lopez-Escarpa D., Pucciarelli M.G., Cestero J.J., Baquero F., Garcia-Del Portillo F. (2020). An alternative penicillin-binding protein involved in *Salmonella* relapses following ceftriaxone therapy. EBioMedicine.

[46] Lewis G.L., Fenton R.J., Moriyama E.N., Loy J.D., Moxley R.A. (2023). Association of ISVsa3 with Multidrug Resistance in *Salmonella* enterica Isolates from Cattle (*Bos taurus*). Microorganisms.

[47] Dolejska M., Villa L., Hasman H., Hansen L., Carattoli A. (2013). Characterization of IncN plasmids carrying bla CTX-M-1 and qnr genes in *Escherichia coli* and *Salmonella* from animals, the environment and humans. J. Antimicrob. Chemother..

[48] Jacoby G.A., Walsh K.E., Mills D.M., Walker V.J., Oh H., Robicsek A., Hooper D.C. (2006). qnrB, another plasmid-mediated gene for quinolone resistance. Antimicrob. Agents Chemother..

[49] Bauernfeind A., Grimm H., Schweighart S. (1990). A new plasmidic cefotaximase in a clinical isolate of *Escherichia coli*. Infection.

[50] Wittum T.E., Mollenkopf D.F., Daniels J.B., Parkinson A.E., Mathews J.L., Fry P.R., Abley M.J., Gebreyes W.A. (2010). CTX-M-type extended-spectrum beta-lactamases present in *Escherichia coli* from the feces of cattle in Ohio, United States. Foodborne Pathog. Dis..

[51] Bauernfeind A.S., Schweighart K. (1990). Dornbusch, and H. Giamarellou. A transfer-able cephamycinase in *Klebsiella pneumoniae*. Program and Abstracts of the 30th Interscience Conference on Antimicrobial Agents and Chemotherapy.

[52] Fey P.D., Safranek T.J., Rupp M.E., Dunne E.F., Ribot E., Iwen P.C., Bradford P.A., Angulo F.J., Hinrichs S.H. (2000). Ceftriaxone-resistant salmonella infection acquired by a child from cattle. N. Engl. J. Med..

[53] Zhao L., Li X., Atwill E.R., Aly S.S., Williams D.R., Su Z. (2022). Dynamic changes in fecal bacterial microbiota of dairy cattle across the production line. BMC Microbiol..

[54] Shade A. (2017). Diversity is the question, not the answer. ISME J..

[55] Wagner R.D., Johnson S.J., Cerniglia C.E., Erickson B.D. (2011). Bovine intestinal bacteria inactivate and degrade ceftiofur and ceftriaxone with multiple beta-lactamases. Antimicrob. Agents Chemother..

[56] Poretsky R., Rodriguez R.L., Luo C., Tsementzi D., Konstantinidis K.T. (2014). Strengths and limitations of 16S rRNA gene amplicon sequencing in revealing temporal microbial community dynamics. PLoS ONE.

[57] Wichmann F., Udikovic-Kolic N., Andrew S., Handelsman J. (2014). Diverse antibiotic resistance genes in dairy cow manure. mBio.

[58] Chambers L., Yang Y., Littier H., Ray P., Zhang T., Pruden A., Strickland M., Knowlton K. (2015). Metagenomic Analysis of Antibiotic Resistance Genes in Dairy Cow Feces following Therapeutic Administration of Third Generation Cephalosporin. PLoS ONE.

