# Supplementary material for: Two-Dose Ceftiofur Treatment Increases Cephamycinase Gene Quantities and Fecal Microbiome Diversity in Dairy Cows Diagnosed with Metritis
Source: Microorganisms. 2023 Nov 8;11(11):2728. doi: 10.3390/microorganisms11112728 (PMC10673576; doi:10.3390/microorganisms11112728)
Supplement: Supplementary file 1 [file microorganisms-11-02728-s001.zip › microorganisms-2666126-supplementary.pdf]

Supplementary Materials

Two-dose ceftiofur treatment increases cephamycinase gene quantities and fecal microbiome diversity in dairy cows diagnosed with metritis

Claudia Ossa-Trujillo[1], Ethan A. Taylor[2], Fatima Sarwar[2], Javier Vinasco[2], Ellen R. Jordan[3], Jose A. García Buitrago[4], G. Robert Hagevoort[4], Sara D. Lawhon[2], Juan M. Piñeiro[5], Jessica Galloway-Peña[2], Keri N. Norman[1,†,*], Harvey Morgan Scott[2,†,*]

[1] Department of Veterinary Integrative Biosciences, College of Veterinary Medicine and Biomedical Sciences, Texas A&M University, College Station, TX, USA; cossat@tamu.edu (C.O.T.)

[2] Department of Veterinary Pathobiology, Texas A&M University, College Station, TX, USA; eataylor2136@gmail.com (E.A.T.); fatimasarwar15@gmail.com (F.S.); jvinasco-torres@cvm.tamu.edu (J.V.T.); slawhon@cvm.tamu.edu (S.D.L.); jgallowaypena@cvm.tamu.edu (J.G.P.)

[3] Department of Animal Science, Texas A&M University, Dallas, TX, USA; txjordanqh@yahoo.com (E.R.J.)

[4] Department of Extension Animal Sciences and Natural Resources, New Mexico State University, Clovis, NM, USA; jarmando@nmsu.edu (J.A.G.); dairydoc@nmsu.edu (G.R.H.)

[5] Department of Animal Science, Texas A&M University, Amarillo, TX, USA; juan.pineiro@ag.tamu.edu (J.M.P.)

* Correspondence: knorman@cvm.tamu.edu (K.N.N.); hmscott@cvm.tamu.edu (H.M.S.)

† These authors contributed equally to this work

Table S1. 744 fecal samples across days 0, 6, and 16, pooled in four groups by dairy farm, day, and treatment (192 fecal pools in total)(labID-DairyTreatment-AnimalID-SampleDay-Season).

Day 0 (Yellow = Missing)											
Treated						Untreated					
	Group 1A-Ca:		Group 1B-Ca:		Group 1C-Ca:		Group 2A-Co:		Group 2B-Co:		Group 2C-Co:
	1-A1-9338-0-1		31-B1-36031-0-1		63-C1-15522-0-1		2-A2-11209-0-1		32-B2-40165-0-1		64-C2-16463-0-1
**1**	3-A1-13028-0-1	**12**	33-B1-40812-0-1	**23**	65-C1-21250-0-1	**33**	4-A2-12908-0-1	**44**	34-B2-40905-0-1	**55**	66-C2-21191-0-1
	5-A1-12929-0-1		35-B1-40735-0-1		67-C1-21211-0-1		6-A2-12984-0-1		36-B2-40705-0-1		68-C2-21041-0-1
	7-A1-12108-0-1		37-B1-40923-0-1		69-C1-16443-0-1		8-A2-12778-0-1		38-B2-40799-0-1		70-C2-18858-0-1
	Group 3A-Ca:		Group 3B-Ca:		Group 3C-Ca:		Group 4A-Co:		Group 4B-Co:		Group 4C-Co:
	9-A1-13233-0-1		39-B1-40983-0-1		71-C1-21276-0-1		10-A2-12975-0-1		40-B2-40978-0-1		72-C2-21004-0-1
**2**	11-A1-10405-0-1	**13**	41-B1-31444-0-1	**24**	73-C1-19111-0-1	**34**	12-A2-10844-0-1	**45**	42-B2-29555-0-1	**56**	74-C2-19003-0-1
	13-A1-10481-0-1		43-B1-33139-0-1		75-C1-19827-0-1		14-A2-6166-0-1		44-B2-33100-0-1		76-C2-19104-0-1
	15-A1-13108-0-1		45-B1-40968-0-1		77-C1-19097-0-1		16-A2-13301-0-1		46-B2-40974-0-1		78-C2-18999-0-1
	Group 5A-Ca:		Group 5B-Ca:		Group 5C-Ca:		Group 6A-Co:		Group 6B-Co:		Group 6C-Co:
	17-A1-13286-0-1		47-B1-40824-0-1		79-C1-8487-0-1		18-A2-12946-0-1		48-B2-40889-0-1		80-C2-18595-0-1
**3**	19-A1-11374-0-1	**14**	49-B1-34376-0-1	**25**	81-C1-21188-0-1	**35**	20-A2-10813-0-1	**46**	50-B2-32452-0-1	**57**	82-C2-20427-0-1
	21-A1-13026-0-1		51-B1-36165-0-1		83-C1-17501-0-1		22-A2-13289-0-1		52-B2-40908-0-1		84-C2-18112-0-1
	23-A1-13302-0-1		53-B1-30994-0-1		85-C1-20201-0-1		24-A2-13137-0-1		54-B2-40843-0-1		86-C2-20322-0-1
	Group 7A-Ca:		Group 7B-Ca:		Group 7C-Ca:		Group 8A-Co:		Group 8B-Co:		Group 8C-Co:
	25-A1-13332-0-1		55-B1-40981-0-1		87-C1-21196-0-1		26-A2-13069-0-1		56-B2-40821-0-1		88-C2-21398-0-1
**4**	27-A1-12917-0-1	**15**	57-B1-40734-0-1	**26**	89-C1-20994-0-1	**36**	28-A2-12954-0-1	**47**	58-B2-38424-0-1	**58**	90-C2-20326-0-1
	29-A1-13217-0-1		59-B1-29166-0-1		91-C1-21035-0-1		30-A2-13095-0-1		60-B2-40631-0-1		92-C2-21148-0-1
	1-A1-10277-0-2		61-B1-44088-0-1		101-C1-19732-0-2		2-A2-54-0-2		62-B2-32193-0-1		102-C2-19822-0-2
	Group 9A-Ca:		Group 9B-Ca:		Group 9C-Ca:		Group 10A-Co:		Group 10B-Co:		Group 10C-Co:
	3-A1-3846-0-2		51-B1-43305-0-2		103-C1-21860-0-2		4-A2-10313-0-2		52-B2-32010-0-2		104-C2-21576-0-2
**5**	5-A1-6098-0-2	**16**	53-B1-43359-0-2	**27**	105-C1-22072-0-2	**37**	6-A2-6772-0-2	**48**	54-B2-41841-0-2	**59**	106-C2-21805-0-2
	7-A1-12585-0-2		55-B1-43367-0-2		107-C1-21096-0-2		8-A2-10132-0-2		56-B2-43028-0-2		108-C2-21822-0-2
	9-A1-14134-0-2		57-B1-43451-0-2		109-C1-21563-0-2		10-A2-13736-0-2		58-B2-43429-0-2		110-C2-21164-0-2
	Group 11A-Ca:		Group 11B-Ca:		Group 11C-Ca:		Group 12A-Co:		Group 12B-Co:		Group 12C-Co:
	11-A1-14165-0-2		59-B1-26908-0-2		111-C1-18488-0-2		12-A2-14000-0-2		60-B2-28765-0-2		112-C2-21673-0-2
**6**	13-A1-13959-0-2	**17**	61-B1-31714-0-2	**28**	113-C1-21857-0-2	**38**	14-A2-12492-0-2	**49**	62-B2-30874-0-2	**60**	114-C2-18071-0-2
	15-A1-13944-0-2		63-B1-37214-0-2		115-C1-22043-0-2		16-A2-14127-0-2		64-B2-41490-0-2		116-C2-21922-0-2
	17-A1-13590-0-2		65-B1-41918-0-2		117-C1-20037-0-2		18-A2-13815-0-2		66-B2-43394-0-2		118-C2-22265-0-2
	Group 13A-Ca:		Group 13B-Ca:		Group 13C-Ca:		Group 14A-Co:		Group 14B-Co:		Group 14C-Co:
	19-A1-14214-0-2		67-B1-37051-0-2		119-C1-21882-0-2		20-A2-13948-0-2		68-B2-37713-0-2		120-C2-21833-0-2
**7**	21-A1-13596-0-2	**18**	69-B1-37890-0-2	**29**	121-C1-22256-0-2	**39**	22-A2-13935-0-2	**50**	70-B2-41095-0-2	**61**	122-C2-845-0-2
	23-A1-13934-0-2		71-B1-43361-0-2		123-C1-19590-0-2		24-A2-14263-0-2		72-B2-43246(1)-0-2		124-C2-19680-0-2
	25-A1-14161-0-2		73-B1-43485-0-2		125-C1-19623-0-2		26-A2-14013-0-2		74-B2-41843-0-2		126-C2-19664-0-2
	Group 15A-Ca:		Group 15B-Ca:		Group 15C-Ca:		Group 16A-Co:		Group 16B-Co:		Group 16C-Co:
	27-A1-12660-0-2		75-B1-36248-0-2		127-C1-20401-0-2		28-A2-11840-0-2		76-B2-33482-0-2		128-C2-19774-0-2
**8**	29-A1-10368-0-2	**19**	77-B1-41813-0-2	**30**	129-C1-22557-0-2	**40**	30-A2-12690-0-2	**51**	78-B2-35559-0-2	**62**	130-C2-22202-0-2
	31-A1-11754-0-2		79-B1-43283-0-2		131-C1-22367-0-2		32-A2-11089-0-2		80-B2-43246(2)-0-2		132-C2-21855-0-2
	33-A1-10604-0-2		81-B1-37962-0-2		133-C1-22604-0-2		34-A2-6770-0-2		82-B2-30763-0-2		134-C2-22136-0-2
	Group 17A-Ca:		Group 17B-Ca:		Group 17C-Ca:		Group 18A-Co:		Group 18B-Co:		Group 18C-Co:
	35-A1-14143-0-2		83-B1-35558-0-2		135-C1-22431-0-2		36-A2-14197-0-2		84-B2-43449-0-2		136-C2-22119-0-2
**9**	37-A1-14782-0-2	**20**	85-B1-43322-0-2	**31**	137-C1-18815-0-2	**41**	38-A2-14307-0-2	**52**	86-B2-41733-0-2	**63**	138-C2-19712-0-2
	39-A1-3082-0-2		87-B1-40510-0-2		139-C1-21990-0-2		40-A2-2654-0-2		88-B2-43835-0-2		140-C2-22560-0-2
	41-A1-10815-0-2		89-B1-46730-0-2		141-C1-21856-0-2		42-A2-12716-0-2		90-B2-44120-0-2		142-C2-21642-0-2
	Group 19A-Ca:		Group 19B-Ca:		Group 19C-Ca:		Group 20A-Co:		Group 20B-Co:		Group 20C-Co:
	43-A1-10677-0-2		91-B1-44003-0-2		143-C1-22028-0-2		44-A2-12458-0-2		92-B2-43986-0-2		144-C2-22384-0-2
**10**	45-A1-12147-0-2	**21**	93-B1-24231-0-2	**32**	145-C1-20208-0-2	**42**	46-A2-9389-0-2	**53**	94-B2-29955-0-2	**64**	146-C2-22568-0-2
	47-A1-14583-0-2		95-B1-43984-0-2		147-C1-21804-0-2		48-A2-14688-0-2		96-B2-38404-0-2		148-C2-21918-0-2
	49-A1-17686-0-2		97-B1-26145-0-2		149-C1-22558-0-2		50-A2-10456-0-2		98-B2-37126-0-2		150-C2-22504-0-2
	Group 21A-Ca:		Group 21B-Ca:				Group 22A-Co:		Group 22B-Co:		
**11**	153-A1-8139-0-2	**22**	99-B1-37198-0-2			**43**	154-A2-4345-0-2	**54**	100-B2-44017-0-2		
	155-A1-14716-0-2		151-B1-31145-0-2				156-A2-14282-0-2		152-B2-30881-0-2		

Table S1. Continuation. The yellow highlight indicates missing feces due to insufficient sample to add to the pool.

Day 6 (Yellow = Missing)											
Treated						Untreated					
	Group 1A-Ca:		Group 1B-Ca:		Group 1C-Ca:		Group 2A-Co:		Group 2B-Co:		Group 2C-Co:
	1-A1-9338-6-1		31-B1-36031-6-1		63-C1-15522-6-1		2-A2-11209-6-1		32-B2-40165-6-1		64-C2-16463-6-1
65	3-A1-13028-6-1	76	33-B1-40812-6-1	87	65-C1-21250-6-1	97	4-A2-12908-6-1	108	34-B2-40905-6-1	119	66-C2-21191-6-1
	5-A1-12929-6-1		35-B1-40735-6-1		67-C1-21211-6-1		6-A2-12984-6-1		36-B2-40705-6-1		68-C2-21041-6-1
	7-A1-12108-6-1		37-B1-40923-6-1		69-C1-16443-6-1		8-A2-12778-6-1		38-B2-40799-6-1		70-C2-18858-6-1
	Group 3A-Ca:		Group 3B-Ca:		Group 3C-Ca:		Group 4A-Co:		Group 4B-Co:		Group 4C-Co:
	9-A1-13233-6-1		39-B1-40983-6-1		71-C1-21276-6-1		10-A2-12975-6-1		40-B2-40978-6-1		72-C2-21004-6-1
66	11-A1-10405-6-1	77	41-B1-31444-6-1	88	73-C1-19111-6-1	98	12-A2-10844-6-1	109	42-B2-29555-6-1	120	74-C2-19003-6-1
	13-A1-10481-6-1		43-B1-33139-6-1		75-C1-19827-6-1		14-A2-6166-6-1		44-B2-33100-6-1		76-C2-19104-6-1
	15-A1-13108-6-1		45-B1-40968-6-1		77-C1-19097-6-1		16-A2-13301-6-1		46-B2-40974-6-1		78-C2-18999-6-1
	Group 5A-Ca:		Group 5B-Ca:		Group 5C-Ca:		Group 6A-Co:		Group 6B-Co:		Group 6C-Co:
	17-A1-13286-6-1		47-B1-40824-6-1		79-C1-8487-6-1		18-A2-12946-6-1		48-B2-40889-6-1		80-C2-18595-6-1
67	19-A1-11374-6-1	78	49-B1-34376-6-1	89	81-C1-21188-6-1	99	20-A2-10813-6-1	110	50-B2-32452-6-1	121	82-C2-20427-6-1
	21-A1-13026-6-1		51-B1-36165-6-1		83-C1-17501-6-1		22-A2-13289-6-1		52-B2-40908-6-1		84-C2-18112-6-1
	23-A1-13302-6-1		53-B1-30994-6-1		85-C1-20201-6-1		24-A2-13137-6-1		54-B2-40843-6-1		86-C2-20322-6-1
	Group 7A-Ca:		Group 7B-Ca:		Group 7C-Ca:		Group 8A-Co:		Group 8B-Co:		Group 8C-Co:
	25-A1-13332-6-1		55-B1-40981-6-1		87-C1-21196-6-1		26-A2-13069-6-1		56-B2-40821-6-1		88-C2-21398-6-1
68	27-A1-12917-6-1	79	57-B1-40734-6-1	90	89-C1-20994-6-1	100	28-A2-12954-6-1	111	58-B2-38424-6-1	122	90-C2-20326-6-1
	29-A1-13217-6-1		59-B1-29166-6-1		91-C1-21035-6-1		30-A2-13095-6-1		60-B2-40631-6-1		92-C2-21148-6-1
	1-A1-10277-6-2		61-B1-44088-6-1		101-C1-19732-6-2		2-A2-54-6-2		62-B2-32193-6-1		102-C2-19822-6-2
	Group 9A-Ca:		Group 9B-Ca:		Group 9C-Ca:		Group 10A-Co:		Group 10B-Co:		Group 10C-Co:
	3-A1-3846-6-2		51-B1-43305-6-2		103-C1-21860-6-2		4-A2-10313-6-2		52-B2-32010-6-2		104-C2-21576-6-2
69	5-A1-6098-6-2	80	53-B1-43359-6-2	91	105-C1-22072-6-2	101	6-A2-6772-6-2	112	54-B2-41841-6-2	123	106-C2-21805-6-2
	7-A1-12585-6-2		55-B1-43367-6-2		107-C1-21096-6-2		8-A2-10132-6-2		56-B2-43028-6-2		108-C2-21822-6-2
	9-A1-14134-6-2		57-B1-43451-6-2		109-C1-21563-6-2		10-A2-13736-6-2		58-B2-43429-6-2		110-C2-21164-6-2
	Group 11A-Ca:		Group 11B-Ca:		Group 11C-Ca:		Group 12A-Co:		Group 12B-Co:		Group 12C-Co:
	11-A1-14165-6-2		59-B1-26908-6-2		111-C1-18488-6-2		12-A2-14000-6-2		60-B2-28765-6-2		112-C2-21673-6-2
70	13-A1-13959-6-2	81	61-B1-31714-6-2	92	113-C1-21857-6-2	102	14-A2-12492-6-2	113	62-B2-30874-6-2	124	114-C2-18071-6-2
	15-A1-13944-6-2		63-B1-37214-6-2		115-C1-22043-6-2		16-A2-14127-6-2		64-B2-41490-6-2		116-C2-21922-6-2
	17-A1-13590-6-2		65-B1-41918-6-2		117-C1-20037-6-2		18-A2-13815-6-2		66-B2-43394-6-2		118-C2-22265-6-2
	Group 13A-Ca:		Group 13B-Ca:		Group 13C-Ca:		Group 14A-Co:		Group 14B-Co:		Group 14C-Co:
	19-A1-14214-6-2		67-B1-37051-6-2		119-C1-21882-6-2		20-A2-13948-6-2		68-B2-37713-6-2		120-C2-21833-6-2
71	21-A1-13596-6-2	82	69-B1-37890-6-2	93	121-C1-22256-6-2	103	22-A2-13935-6-2	114	70-B2-41095-6-2	125	122-C2-845-6-2
	23-A1-13934-6-2		71-B1-43361-6-2		123-C1-19590-6-2		24-A2-14263-6-2		72-B2-43246(1)-6-2		124-C2-19680-6-2
	25-A1-14161-6-2		73-B1-43485-6-2		125-C1-19623-6-2		26-A2-14013-6-2		74-B2-41843-6-2		126-C2-19664-6-2
	Group 15A-Ca:		Group 15B-Ca:		Group 15C-Ca:		Group 16A-Co:		Group 16B-Co:		Group 16C-Co:
	27-A1-12660-6-2		75-B1-36248-6-2		127-C1-20401-6-2		28-A2-11840-6-2		76-B2-33482-6-2		128-C2-19774-6-2
72	29-A1-10368-6-2	83	77-B1-41813-6-2	94	129-C1-22557-6-2	104	30-A2-12690-6-2	115	78-B2-35559-6-2	126	130-C2-22202-6-2
	31-A1-11754-6-2		79-B1-43283-6-2		131-C1-22367-6-2		32-A2-11089-6-2		80-B2-43246(2)-6-2		132-C2-21855-6-2
	33-A1-10604-6-2		81-B1-37962-6-2		133-C1-22604-6-2		34-A2-6770-6-2		82-B2-30763-6-2		134-C2-22136-6-2
	Group 17A-Ca:		Group 17B-Ca:		Group 17C-Ca:		Group 18A-Co:		Group 18B-Co:		Group 18C-Co:
	35-A1-14143-6-2		83-B1-35558-6-2		135-C1-22431-6-2		36-A2-14197-6-2		84-B2-43449-6-2		136-C2-22119-6-2
73	37-A1-14782-6-2	84	85-B1-43322-6-2	95	137-C1-18815-6-2	105	38-A2-14307-6-2	116	86-B2-41733-6-2	127	138-C2-19712-6-2
	39-A1-3082-6-2		87-B1-40510-6-2		139-C1-21990-6-2		40-A2-2654-6-2		88-B2-43835-6-2		140-C2-22560-6-2
	41-A1-10815-6-2		89-B1-46730-6-2		141-C1-21856-6-2		42-A2-12716-6-2		90-B2-44120-6-2		142-C2-21642-6-2
	Group 19A-Ca:		Group 19B-Ca:		Group 19C-Ca:		Group 20A-Co:		Group 20B-Co:		Group 20C-Co:
	43-A1-10677-6-2		91-B1-44003-6-2		143-C1-22028-6-2		44-A2-12458-6-2		92-B2-43986-6-2		144-C2-22384-6-2
74	45-A1-12147-6-2	85	93-B1-24231-6-2	96	145-C1-20208-6-2	106	46-A2-9389-6-2	117	94-B2-29955-6-2	128	146-C2-22568-6-2
	47-A1-14583-6-2		95-B1-43984-6-2		147-C1-21804-6-2		48-A2-14688-6-2		96-B2-38404-6-2		148-C2-21918-6-2
	49-A1-17686-6-2		97-B1-26145-6-2		149-C1-22558-6-2		50-A2-10456-6-2		98-B2-37126-6-2		150-C2-22504-6-2
	Group 21A:		Group 21B-Ca:				Group 22A-Co:		Group 22B-Co:		
75	153-A1-8139-6-2	86	99-B1-37198-6-2			107	154-A2-4345-6-2	118	100-B2-44017-6-2		
	155-A1-14716-6-2		151-B1-31145-6-2				156-A2-14282-6-2		152-B2-30881-6-2		

Table S1. Continuation. The yellow highlight indicates missing feces due to insufficient sample to add to the pool.

Day 16 (Yellow = Missing)											
Treated						Untreated					
	Group 1A-Ca:		Group 1B-Ca:		Group 1C-Ca:		Group 2A-Co:		Group 2B-Co:		Group 2C-Co:
	1-A1-9338-16-1		31-B1-36031-16-1		63-C1-15522-16-1		2-A2-11209-16-1		32-B2-40165-16-1		64-C2-16463-16-1
**129**	3-A1-13028-16-1	**140**	33-B1-40812-16-1	**151**	65-C1-21250-16-1	**161**	4-A2-12908-16-1	**172**	34-B2-40905-16-1	**183**	66-C2-21191-16-1
	5-A1-12929-16-1		35-B1-40735-16-1		67-C1-21211-16-1		6-A2-12984-16-1		36-B2-40705-16-1		68-C2-21041-16-1
	7-A1-12108-16-1		37-B1-40923-16-1		69-C1-16443-16-1		8-A2-12778-16-1		38-B2-40799-16-1		70-C2-18858-16-1
	Group 3A-Ca:		Group 3B-Ca:		Group 3C-Ca:		Group 4A-Co:		Group 4B-Co:		Group 4C-Co:
	9-A1-13233-16-1		39-B1-40983-16-1		71-C1-21276-16-1		10-A2-12975-16-1		40-B2-40978-16-1		72-C2-21004-16-1
**130**	11-A1-10405-16-1	**141**	41-B1-31444-16-1	**152**	73-C1-19111-16-1	**162**	12-A2-10844-16-1	**173**	42-B2-29555-16-1	**184**	74-C2-19003-16-1
	13-A1-10481-16-1		43-B1-33139-16-1		75-C1-19827-16-1		14-A2-6166-16-1		44-B2-33100-16-1		76-C2-19104-16-1
	15-A1-13108-16-1		45-B1-40968-16-1		77-C1-19097-16-1		16-A2-13301-16-1		46-B2-40974-16-1		78-C2-18999-16-1
	Group 5A-Ca:		Group 5B-Ca:		Group 5C-Ca:		Group 6A-Co:		Group 6B-Co:		Group 6C-Co:
	17-A1-13286-16-1		47-B1-40824-16-1		79-C1-8487-16-1		18-A2-12946-16-1		48-B2-40889-16-1		80-C2-18595-16-1
**131**	19-A1-11374-16-1	**142**	49-B1-34376-16-1	**153**	81-C1-21188-16-1	**163**	20-A2-10813-16-1	**174**	50-B2-32452-16-1	**185**	82-C2-20427-16-1
	21-A1-13026-16-1		51-B1-36165-16-1		83-C1-17501-16-1		22-A2-13289-16-1		52-B2-40908-16-1		84-C2-18112-16-1
	23-A1-13302-16-1		53-B1-30994-16-1		85-C1-20201-16-1		24-A2-13137-16-1		54-B2-40843-16-1		86-C2-20322-16-1
	Group 7A-Ca:		Group 7B-Ca:		Group 7C-Ca:		Group 8A-Co:		Group 8B-Co:		Group 8C-Co:
	25-A1-13332-16-1		55-B1-40981-16-1		87-C1-21196-16-1		26-A2-13069-16-1		56-B2-40821-16-1		88-C2-21398-16-1
**132**	27-A1-12917-16-1	**143**	57-B1-40734-16-1	**154**	89-C1-20994-16-1	**164**	28-A2-12954-16-1	**175**	58-B2-38424-16-1	**186**	90-C2-20326-16-1
	29-A1-13217-16-1		59-B1-29166-16-1		91-C1-21035-16-1		30-A2-13095-16-1		60-B2-40631-16-1		92-C2-21148-16-1
	1-A1-10277-16-2		61-B1-44088-16-1		101-C1-19732-16-2		2-A2-54-16-2		62-B2-32193-16-1		102-C2-19822-16-2
	Group 9A-Ca:		Group 9B-Ca:		Group 9C-Ca:		Group 10A-Co:		Group 10B-Co:		Group 10C-Co:
	3-A1-3846-16-2		51-B1-43305-16-2		103-C1-21860-16-2		4-A2-10313-16-2		52-B2-32010-16-2		104-C2-21576-16-2
**133**	5-A1-6098-16-2	**144**	53-B1-43359-16-2	**155**	105-C1-22072-16-2	**165**	6-A2-6772-16-2	**176**	54-B2-41841-16-2	**187**	106-C2-21805-16-2
	7-A1-12585-16-2		55-B1-43367-16-2		107-C1-21096-16-2		8-A2-10132-16-2		56-B2-43028-16-2		108-C2-21822-16-2
	9-A1-14134-16-2		57-B1-43451-16-2		109-C1-21563-16-2		10-A2-13736-16-2		58-B2-43429-16-2		110-C2-21164-16-2
	Group 11A-Ca:		Group 11B-Ca:		Group 11C-Ca:		Group 12A-Co:		Group 12B-Co:		Group 12C-Co:
	11-A1-14165-16-2		59-B1-26908-16-2		111-C1-18488-16-2		12-A2-14000-16-2		60-B2-28765-16-2		112-C2-21673-16-2
**134**	13-A1-13959-16-2	**145**	61-B1-31714-16-2	**156**	113-C1-21857-16-2	**166**	14-A2-12492-16-2	**177**	62-B2-30874-16-2	**188**	114-C2-18071-16-2
	15-A1-13944-16-2		63-B1-37214-16-2		115-C1-22043-16-2		16-A2-14127-16-2		64-B2-41490-16-2		116-C2-21922-16-2
	17-A1-13590-16-2		65-B1-41918-16-2		117-C1-20037-16-2		18-A2-13815-16-2		66-B2-43394-16-2		118-C2-22265-16-2
	Group 13A-Ca:		Group 13B-Ca:		Group 13C-Ca:		Group 14A-Co:		Group 14B-Co:		Group 14C-Co:
	19-A1-14214-16-2		67-B1-37051-16-2		119-C1-21882-16-2		20-A2-13948-16-2		68-B2-37713-16-2		120-C2-21833-16-2
**135**	21-A1-13596-16-2	**146**	69-B1-37890-16-2	**157**	121-C1-22256-16-2	**167**	22-A2-13935-16-2	**178**	70-B2-41095-16-2	**189**	122-C2-845-16-2
	23-A1-13934-16-2		71-B1-43361-16-2		123-C1-19590-16-2		24-A2-14263-16-2		72-B2-43246(1)-16-2		124-C2-19680-16-2
	25-A1-14161-16-2		73-B1-43485-16-2		125-C1-19623-16-2		26-A2-14013-16-2		74-B2-41843-16-2		126-C2-19664-16-2
	Group 15A-Ca:		Group 15B-Ca:		Group 15C-Ca:		Group 16A-Co:		Group 16B-Co:		Group 16C-Co:
	27-A1-12660-16-2		75-B1-36248-16-2		127-C1-20401-16-2		28-A2-11840-16-2		76-B2-33482-16-2		128-C2-19774-16-2
**136**	29-A1-10368-16-2	**147**	77-B1-41813-16-2	**158**	129-C1-22557-16-2	**168**	30-A2-12690-16-2	**179**	78-B2-35559-16-2	**190**	130-C2-22202-16-2
	31-A1-11754-16-2		79-B1-43283-16-2		131-C1-22367-16-2		32-A2-11089-16-2		80-B2-43246(2)-16-2		132-C2-21855-16-2
	33-A1-10604-16-2		81-B1-37962-16-2		133-C1-22604-16-2		34-A2-6770-16-2		82-B2-30763-16-2		134-C2-22136-16-2
	Group 17A-Ca:		Group 17B-Ca:		Group 17C-Ca:		Group 18A-Co:		Group 18B-Co:		Group 18C-Co:
	35-A1-14143-16-2		83-B1-35558-16-2		135-C1-22431-16-2		36-A2-14197-16-2		84-B2-43449-16-2		136-C2-22119-16-2
**137**	37-A1-14782-16-2	**148**	85-B1-43322-16-2	**159**	137-C1-18815-16-2	**169**	38-A2-14307-16-2	**180**	86-B2-41733-16-2	**191**	138-C2-19712-16-2
	39-A1-3082-16-2		87-B1-40510-16-2		139-C1-21990-16-2		40-A2-2654-16-2		88-B2-43835-16-2		140-C2-22560-16-2
	41-A1-10815-16-2		89-B1-46730-16-2		141-C1-21856-16-2		42-A2-12716-16-2		90-B2-44120-16-2		142-C2-21642-16-2
	Group 19A-Ca:		Group 19B-Ca:		Group 19C-Ca:		Group 20A-Co:		Group 20B-Co:		Group 20C-Co:
	43-A1-10677-16-2		91-B1-44003-16-2		143-C1-22028-16-2		44-A2-12458-16-2		92-B2-43986-16-2		144-C2-22384-16-2
**138**	45-A1-12147-16-2	**149**	93-B1-24231-16-2	**160**	145-C1-20208-16-2	**170**	46-A2-9389-16-2	**181**	94-B2-29955-16-2	**192**	146-C2-22568-16-2
	47-A1-14583-16-2		95-B1-43984-16-2		147-C1-21804-16-2		48-A2-14688-16-2		96-B2-38404-16-2		148-C2-21918-16-2
	49-A1-17686-16-2		97-B1-26145-16-2		149-C1-22558-16-2		50-A2-10456-16-2		98-B2-37126-16-2		150-C2-22504-16-2
	Group 21A-Ca:		Group 21B-Ca:				Group 22A-Co:		Group 22B-Co:		
**139**	153-A1-8139-16-2	**150**	99-B1-37198-16-2			**171**	154-A2-4345-16-2	**182**	100-B2-44017-16-2		
	155-A1-14716-16-2		151-B1-31145-16-2				156-A2-14282-16-2		152-B2-30881-16-2		

Table S2. *E. coli* isolates previously sequenced that harbored the target genes used as positive controls and templates for qPCR standard curve generation.

NCBI Biosample ID	MALDI-TOF	*qnrB* gene	*bla*$_{CMY}$ gene	*bla*$_{CTX-M}$ gene	*mphA* gene
SAMN14842436	*Escherichia coli*	*qnrB19*	*bla*$_{CMY}$	*bla*$_{CTX-M}$	*mphA*
SAMN14842437	*Escherichia coli*	*qnrB19*	-	*bla*$_{CTX-M}$	*mphA*

Table S3. DNA extracted from the fecal samples distributed across the day, dairy, and treatment in 5 PCR plates per gene.

**PLATE 1**						
	1-2	3-4	5-6	7-8	9-10	11-12
A	1.00E+05	1A-Ca-0	9A-Ca-0	4A-Co-6	12A-Co-6	7A-Ca-16
B	1.00E+04	2A-Co-0	10A-Co-0	5A-Ca-6	13A-Ca-6	8A-Co-16
C	1.00E+03	3A-Ca-0	11A-Ca-0	6A-Co-6	1A-Ca-16	9A-Ca-16
D	1.00E+02	4A-Co-0	12A-Co-0	7A-Ca-6	2A-Co-16	10A-Co-16
E	1.01E+01	5A-Ca-0	13A-Ca-0	8A-Co-6	3A-Ca-16	11A-Ca-16
F	1.00E+00	6A-Co-0	1A-Ca-6	9A-Ca-6	4A-Co-16	12A-Co-16
G	PTC	7A-Ca-0	2A-Co-6	10A-Co-6	5A-Ca-16	13A-Ca-16
H	NTC	8A-Co-0	3A-Ca-6	11A-Ca-6	6A-Co-16	

**PLATE 2**						
	1-2	3-4	5-6	7-8	9-10	11-12
A	1.00E+05	14A-Co-0	22A-Co-0	17A-Ca-6	3B-Ca-6	20A-Co-16
B	1.00E+04	15A-Ca-0	1B-Ca-0	18A-Co-6	4B-Co-6	21A-Ca-16
C	1.00E+03	16A-Co-0	2B-Co-0	19A-Ca-6	14A-Co-16	22A-Co-16
D	1.00E+02	17A-Ca-0	3B-Ca-0	20A-Co-6	15A-Ca-16	1B-Ca-16
E	1.01E+01	18A-Co-0	4B-Co-0	21A-Ca-6	16A-Co-16	2B-Co-16
F	1.00E+00	19A-Ca-0	14A-Co-6	22A-Co-6	17A-Ca-16	3B-Ca-16
G	PTC	20A-Co-0	15A-Ca-6	1B-Ca-6	18A-Co-16	4B-Co-16
H	NTC	21A-Ca-0	16A-Co-6	2B-Co-6	19A-Ca-16	

**PLATE 3**						
	1-2	3-4	5-6	7-8	9-10	11-12
A	1.00E+05	5B-Ca-0	13B-Ca-0	8B-Co-6	16B-Co-6	11B-Ca-16
B	1.00E+04	6B-Co-0	14B-Co-0	9B-Ca-6	17B-Ca-6	12B-Co-16
C	1.00E+03	7B-Ca-0	15B-Ca-0	10B-Co-6	5B-Ca-16	13B-Ca-16
D	1.00E+02	8B-Co-0	16B-Co-0	11B-Ca-6	6B-Co-16	14B-Co-16
E	1.01E+01	9B-Ca-0	17B-Ca-0	12B-Co-6	7B-Ca-16	15B-Ca-16
F	1.00E+00	10B-Co-0	5B-Ca-6	13B-Ca-6	8B-Co-16	16B-Co-16
G	PTC	11B-Ca-0	6B-Co-6	14B-Co-6	9B-Ca-16	17B-Ca-16
H	NTC	12B-Co-0	7B-Ca-6	15B-Ca-6	10B-Co-16	

**PLATE 4**						
	1-2	3-4	5-6	7-8	9-10	11-12
A	1.00E+05	18B-Co-0	4C-Co-0	21B-Ca-6	7C-Ca-6	2C-Co-16
B	1.00E+04	19B-Ca-0	5C-Ca-0	22B-Co-6	8C-Co-6	3C-Ca-16
C	1.00E+03	20B-Co-0	6C-Co-0	1C-Ca-6	18B-Co-16	4C-Co-16
D	1.00E+02	21B-Ca-0	7C-Ca-0	2C-Co-6	19B-Ca-16	5C-Ca-16
E	1.01E+01	22B-Co-0	8C-Co-0	3C-Ca-6	20B-Co-16	6C-Co-16
F	1.00E+00	1C-Ca-0	18B-Co-6	4C-Co-6	21B-Ca-16	7C-Ca-16
G	PTC	2C-Co-0	19B-Ca-6	5C-Ca-6	22B-Co-16	8C-Co-16
H	NTC	3C-Ca-0	20B-Co-6	6C-Co-6	1C-Ca-16	

**PLATE 5**						
	1-2	3-4	5-6	7-8	9-10	11-12
A	1.00E+05	9C-Ca-0	17C-Ca-0	13C-Ca-6	9C-Ca-16	17C-Ca-16
B	1.00E+04	10C-Co-0	18C-Co-0	14C-Co-6	10C-Co-16	18C-Co-16
C	1.00E+03	11C-Ca-0	19C-Ca-0	15C-Ca-6	11C-Ca-16	19C-Ca-16
D	1.00E+02	12C-Co-0	20C-Co-0	16C-Co-6	12C-Co-16	20C-Co-16
E	1.01E+01	13C-Ca-0	9C-Ca-6	17C-Ca-6	13C-Ca-16	
F	1.00E+00	14C-Co-0	10C-Co-6	18C-Co-6	14C-Co-16	
G	PTC	15C-Ca-0	11C-Ca-6	19C-Ca-6	15C-Ca-16	
H	NTC	16C-Co-0	12C-Co-6	20C-Co-6	16C-Co-16	

Table S4. Fecal sample dilution and sample loss during the DNA extraction process.

Procedure during DNA extraction	added	total volume(μl)	factor
1g of fecal sample		1000	
250 mg feces		250	4
Added 0.8ml (800μl) CD1 buffer	800	1050	1
Transfer 0.6ml (600ul) into a new tube		600	1.75
Added 0.2ml (200μl) CD2 buffer	200	800	1
Transfer 0.7ml (700ul) into a new tube		700	1.14
Added 0.6ml (600μl) CD3 buffer	600	1300	1
Transfer 1.3ml (1300ul) into a column		1300	1
Added 0.5ml (500μl) EA buffer	500	1800	1
Added 0.5ml (500μl) C5 buffer	500	2300	1
Elution of DNA (100 ul) C6 buffer by 1min centrifuge		100	1
Take 2ul for the PCR reaction		2	50
			400

Table S5. DNA distribution from the fecal samples across the day, dairy farm, and treatment on two different sequencing runs.

PLATE 1

	1	2	3	4	5	6	7	6	9	10	11	12
A	1A-Ca-0	1A-Ca-6	1A-Ca-16	1B-Ca-0	1B-Ca-6	1B-Ca-16	1C-Ca-0	1C-Ca-6	1C-Ca-16	9A-Ca-0	9A-Ca-6	9A-Ca-16
B	2A-Co-0	2A-Co-6	2A-Co-16	2B-Co-0	2B-Co-6	2B-Co-16	2C-Co-0	2C-Co-6	2C-Co-16	9B-Ca-0	9B-Ca-6	9B-Ca-16
C	3A-Ca-0	3A-Ca-6	3A-Ca-16	3B-Ca-0	3B-Ca-6	3B-Ca-16	3C-Ca-0	3C-Ca-6	3C-Ca-16	9C-Ca-0	9C-Ca-6	9C-Ca-16
D	4A-Co-0	4A-Co-6	4A-Co-16	4B-Co-0	4B-Co-6	4B-Co-16	4C-Co-0	4C-Co-6	4C-Co-16	10A-Co-0	10A-Co-6	10A-Co-16
E	5A-Ca-0	5A-Ca-6	5A-Ca-16	5B-Ca-0	5B-Ca-6	5B-Ca-16	5C-Ca-0	5C-Ca-6	5C-Ca-16	10B-Co-0	10B-Co-6	10B-Co-16
F	6A-Co-0	6A-Co-6	6A-Co-16	6B-Co-0	6B-Co-6	6B-Co-16	6C-Co-0	6C-Co-6	6C-Co-16	10C-Co-0	10C-Co-6	10C-Co-16
G	7A-Ca-0	7A-Ca-6	7A-Ca-16	7B-Ca-0	7B-Ca-6	7B-Ca-16	7C-Ca-0	7C-Ca-6	7C-Ca-16	21A-Ca-0	21A-Ca-6	21A-Ca-16
H	8A-Co-0	8A-Co-6	8A-Co-16	8B-Co-0	8B-Co-6	8B-Co-16	8C-Co-0	8C-Co-6	8C-Co-16	21B-Ca-0	21B-Ca-6	21B-Ca-16

PLATE 2

	1	2	3	4	5	6	7	6	9	10	11	12
A	13A-Ca-0	13A-Ca-6	13A-Ca-16	13B-Ca-0	13B-Ca-6	13B-16	13C-Ca-0	13C-Ca-6	13C-Ca-16	11A-Ca-0	11A-Ca-6	11A-Ca-16
B	14A-Co-0	14A-Co-6	14A-Co-16	14B-Co-0	14B-Co-6	14B-16	14C-Co-0	14C-Co-6	14C-Co-16	11B-Ca-0	11B-Ca-6	11B-Ca-16
C	15A-Ca-0	15A-Ca-6	15A-Ca-16	15B-Ca-0	15B-Ca-6	15B-16	15C-Ca-0	15C-Ca-6	15C-Ca-16	11C-Ca-0	11C-Ca-6	11C-Ca-16
D	16A-Co-0	16A-Co-6	16A-Co-16	16B-Co-0	16B-Co-6	16B-16	16C-Co-0	16C-Co-6	16C-Co-16	12A-Co-0	12A-Co-6	12A-Co-16
E	17A-Ca-0	17A-Ca-6	17A-Ca-16	17B-Ca-0	17B-Ca-6	17B-16	17C-Ca-0	17C-Ca-6	17C-Ca-16	12B-Co-0	12B-Co-6	12B-Co-16
F	18A-Co-0	18A-Co-6	18A-Co-16	18B-Co-0	18B-Co-6	18B-16	18C-Co-0	18C-Co-6	18C-Co-16	12C-Co-0	12C-Co-6	12C-Co-16
G	19A-Ca-0	19A-Ca-6	19A-Ca-16	19B-Ca-0	19B-Ca-6	19B-16	19C-Ca-0	19C-Ca-6	19C-Ca-16	22A-Co-0	22A-Co-6	22A-Co-16
H	20A-Co-0	20A-Co-6	20A-Co-16	20B-Co-0	20B-Co-6	20B-16	20C-Co-0	20C-Co-6	20C-Co-16	22B-Co-0	22B-Co-6	22B-Co-16

Table S6. Descriptive statistics on the distribution of non-standardized gene detections across treatment and sampling days.

Growth metric (Log_{10})	Treatment	Descriptive Statistics	Day		
			0	6	16
bla CMY-2	Treated	Sample Size	51	64	63
		Mean	4.398	5.094	5.355
		Standard Error	0.155	0.079	0.089
		95% CI	4.093-4.704	4.939-5.249	5.18-5.529
		Median	4.355	5.098	5.282
	Untreated	Sample Size	52	54	64
		Mean	4.443	4.332	4.618
		Standard Error	0.134	0.112	0.103
		95% CI	4.179-4.708	4.111-4.553	4.416-4.82
		Median	4.380	4.311	4.700
bla CTX-M	Treated	Sample Size	57	60	62
		Mean	4.130	4.290	4.004
		Standard Error	0.102	0.106	0.087
		95% CI	3.929-4.331	4.082-4.497	3.834-4.175
		Median	4.086	4.268	3.952
	Untreated	Sample Size	56	56	61
		Mean	4.285	4.137	4.183
		Standard Error	0.091	0.104	0.102
		95% CI	4.107-4.464	3.933-4.341	3.983-4.383
		Median	4.392	4.145231	4.106
mphA	Treated	Sample Size	54	58	60
		Mean	5.483	5.329	5.305
		Standard Error	0.093	0.080	0.107
		95% CI	5.666-5.666	5.486-5.486	5.516-5.516
		Median	5.465	5.285	5.087
	Untreated	Sample Size	60	58	56
		Mean	5.475	5.336	5.508
		Standard Error	0.078	0.091	0.126
		95% CI	5.628-5.628	5.515-5.515	5.755-5.755
		Median	5.407	5.188	5.243
qnrB19	Treated	Sample Size	63	64	64
		Mean	4.614	4.372	4.581
		Standard Error	0.090	0.090	0.122
		95% CI	4.437-4.79	4.196-4.548	4.341-4.822
		Median	4.624	4.366	4.365
	Untreated	Sample Size	64	64	64
		Mean	4.738	4.333	4.572
		Standard Error	0.104	0.103	0.114
		95% CI	4.533-4.942	4.132-4.535	4.347-4.797
		Median	4.804	4.280	4.265
16S rRNA	Treated	Sample Size	64	64	64
		Mean	10.518	10.213	10.406
		Standard Error	0.058	0.043	0.082
		95% CI	10.404-10.632	10.128-10.298	10.245-10.567
		Median	10.667	10.318	10.516
	Untreated	Sample Size	64	64	64
		Mean	10.629	10.325	10.592
		Standard Error	0.038	0.069	0.062
		95% CI	10.553-10.704	10.189-10.46	10.471-10.713
		Median	10.667	10.505	10.651

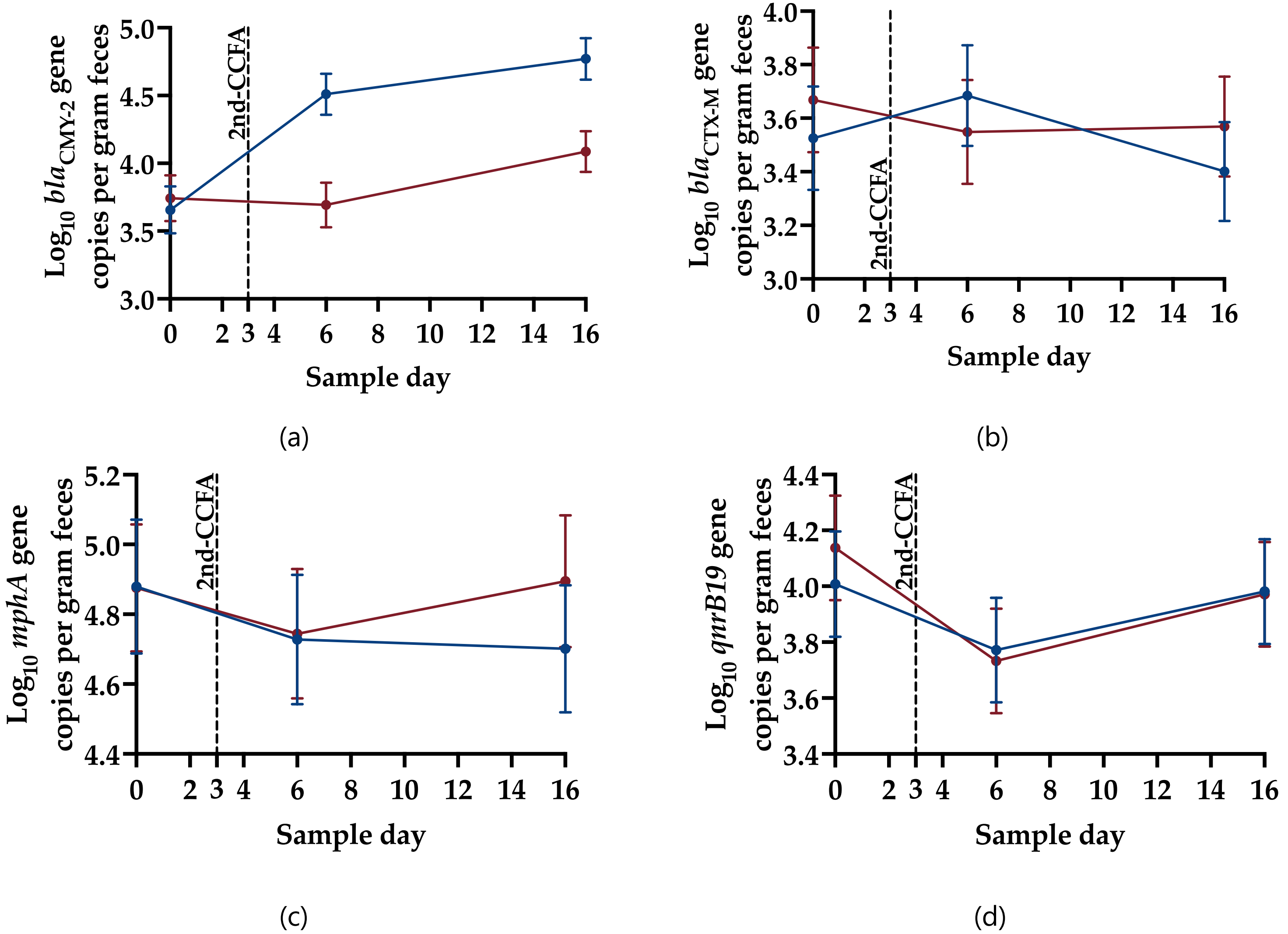

Figure S1. Marginal means graphs showing the linear regression model of non-standardized $\log_{10}$ genes copies per gram of feces based on (a) *bla*$_{\text{CMY-2}}$, (b) *bla*$_{\textit{CTX-M}}$, (c) *mphA*, and (d) *qnrB19*. Data were analyzed using Stata/BE version 17.0 and graphed using GraphPad Prism version 10.0.2.

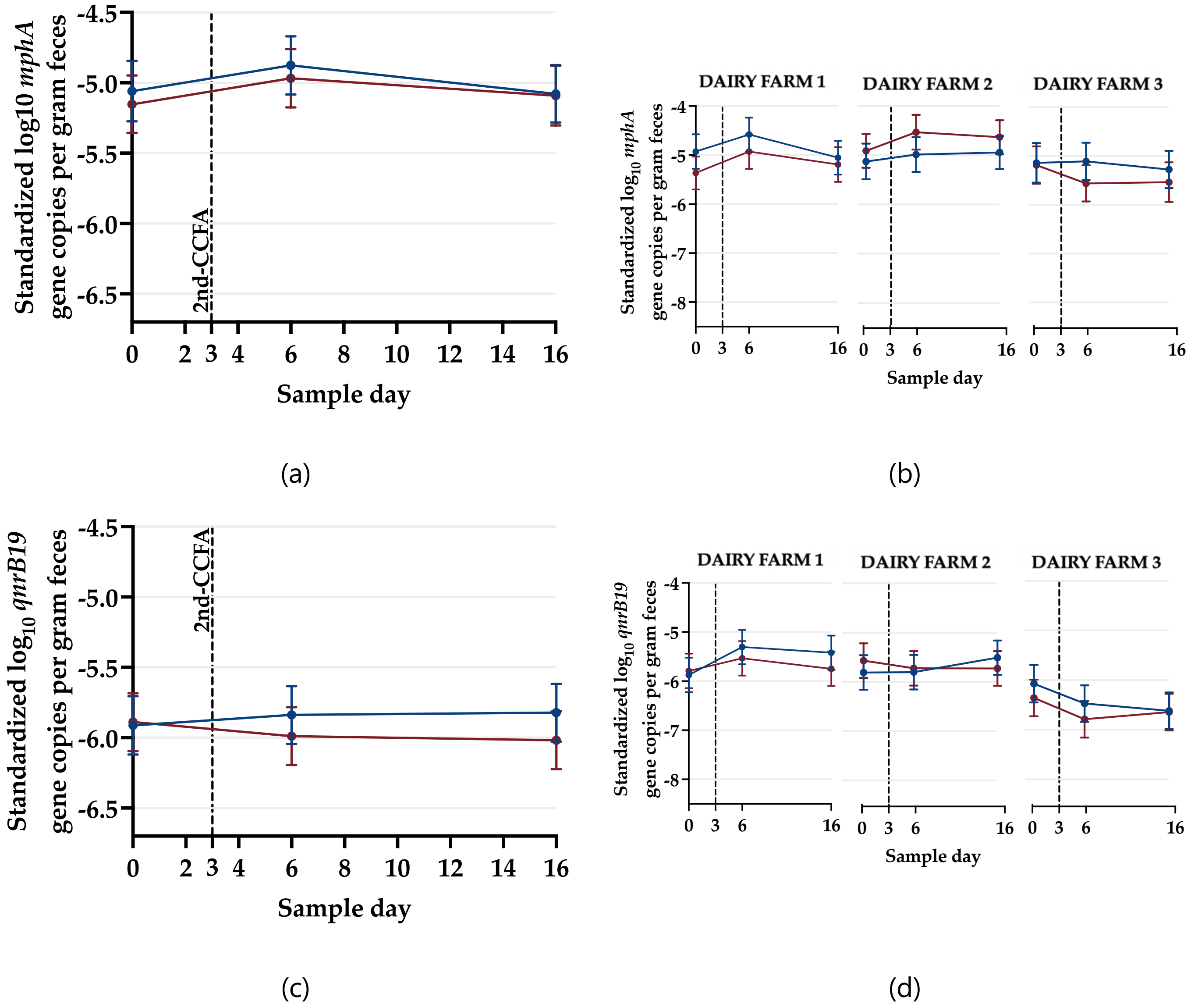

Standardized log10 *mphA* gene copies per gram feces
-4.5
-5.0
-5.5
-6.0
-6.5
2nd-CCFA
0 2 3 4 6 8 10 12 14 16
Sample day
(a)
DAIRY FARM 1
DAIRY FARM 2
DAIRY FARM 3
Standardized log_{10} *mphA* gene copies per gram feces
-4 -5 -6 -7 -8
0 3 6 16
Sample day
(b)
Standardized log_{10} *qnrB19* gene copies per gram feces
-4.5
-5.0
-5.5
-6.0
-6.5
2nd-CCFA
0 2 3 4 6 8 10 12 14 16
Sample day
(c)
DAIRY FARM 1
DAIRY FARM 2
DAIRY FARM 3
Standardized log_{10} *qnrB19* gene copies per gram feces
-4 -5 -6 -7 -8
0 3 6 16
Sample day
(d)

Figure S2. Marginal means graphs showing the linear effect model of 16S rRNA-standardized log_{10} genes copies per gram of feces based on (a) *mphA* and (b) *mphA* by farm. (a) *qnrB19*, and (b) *qnrB19* by farm. Data were analyzed using Stata/BE version 17.0 and graphed using GraphPad Prism version 10.0.2.

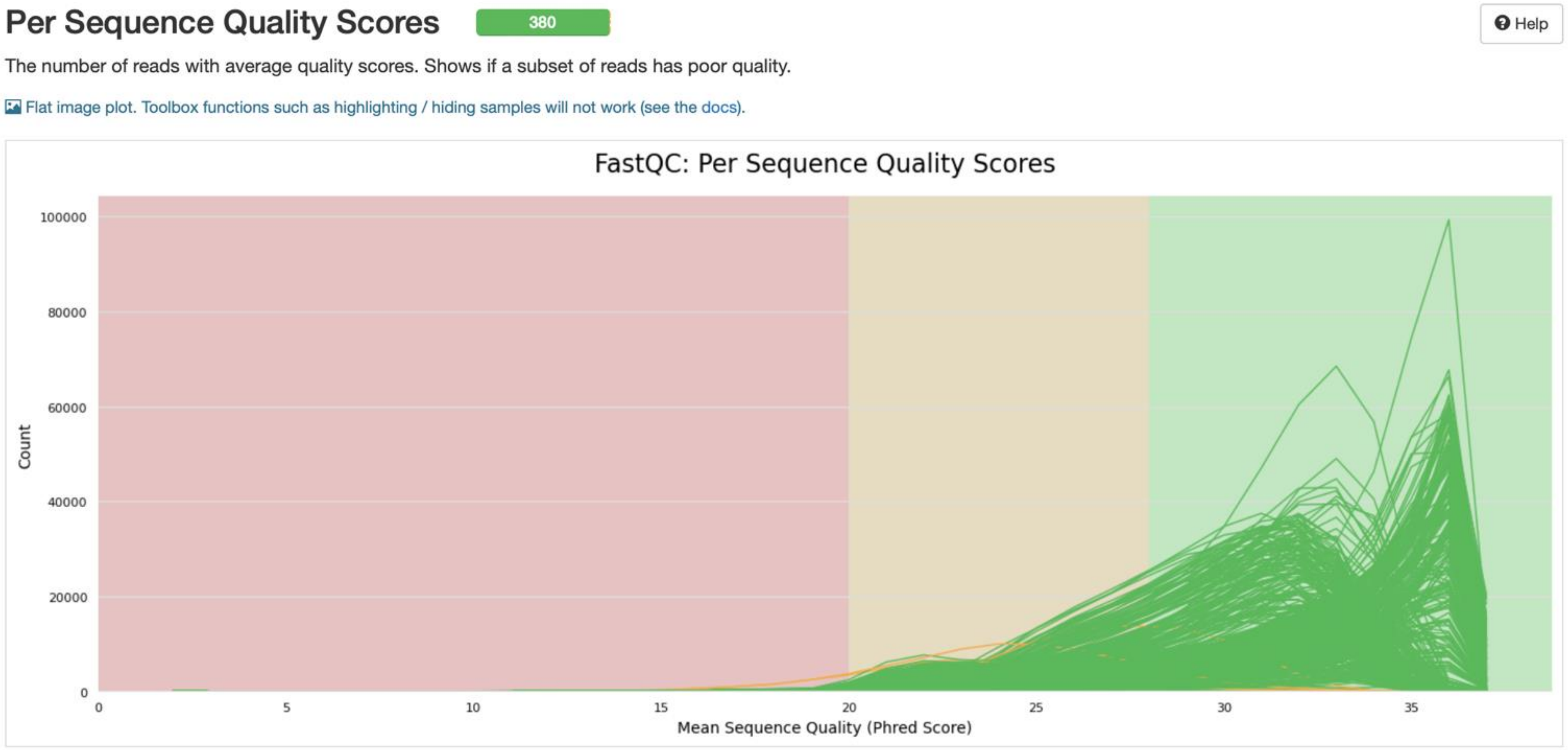

Figure S3. Per sequences quality score from MultiQC output. Graphic produced by MultiQC.

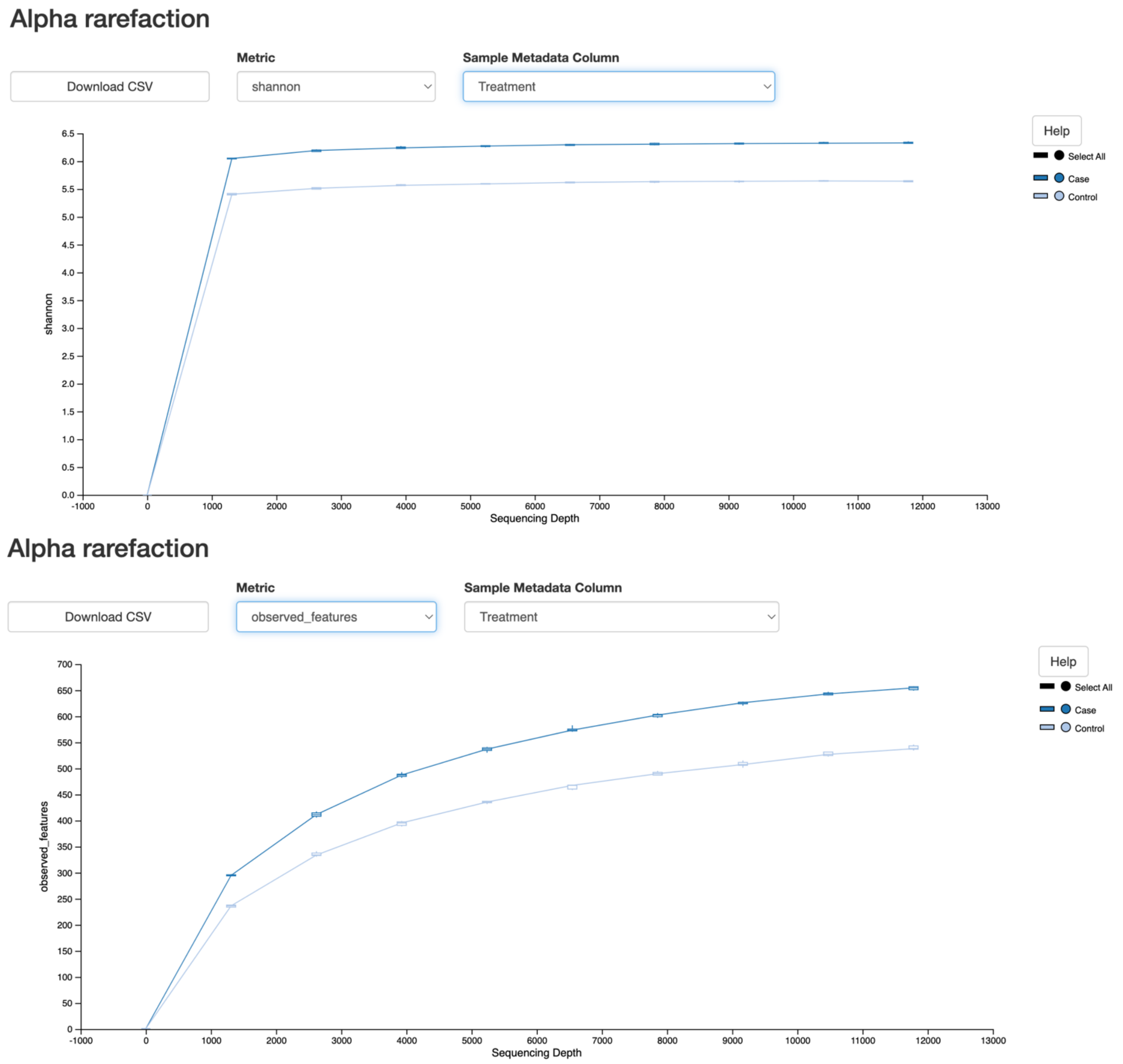

Figure S4. Rarefaction curves displayed by treatment.

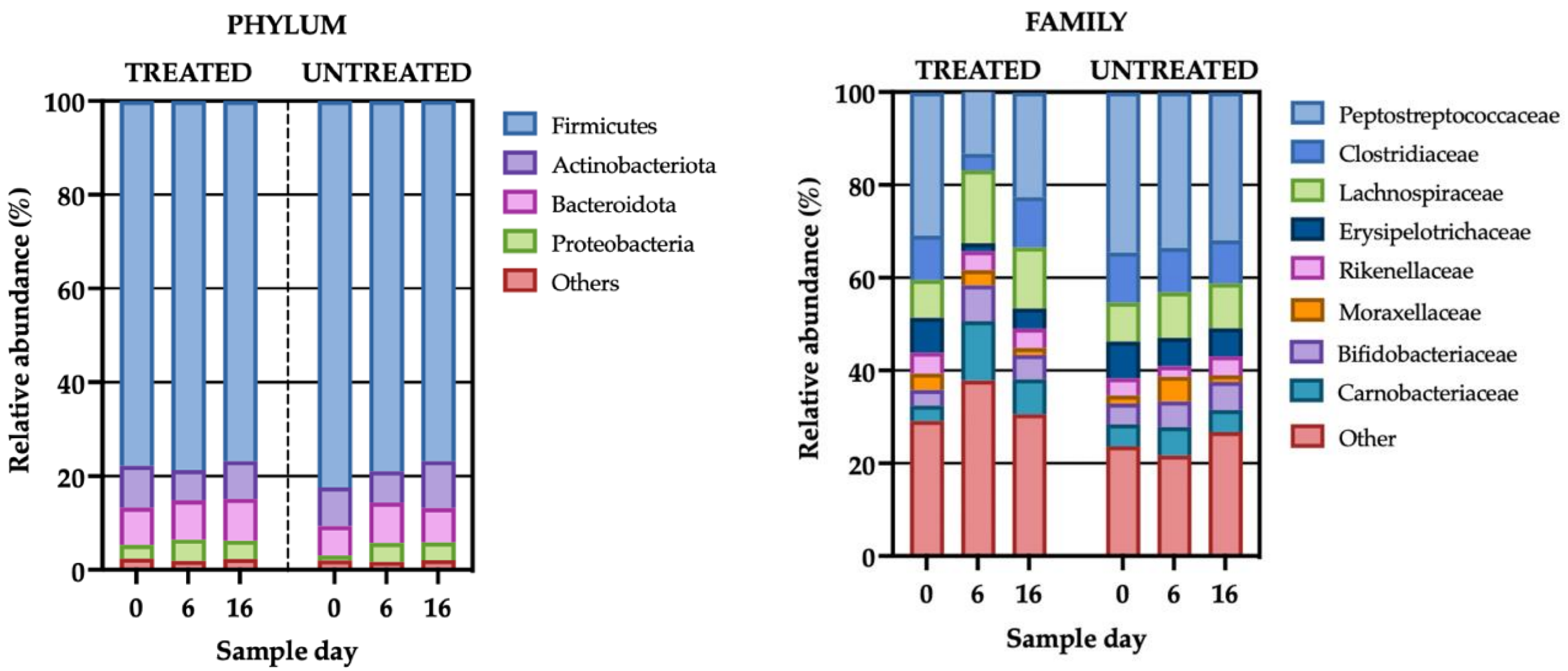

Figure S5. Fecal microbiome relative abundance phylogenetic profile by day and treatment. Comparisons of relative abundance within the bacteria domain at the level of bacterial phylum and family. Graphs were generated using GraphPad Prism version 10.0.2.

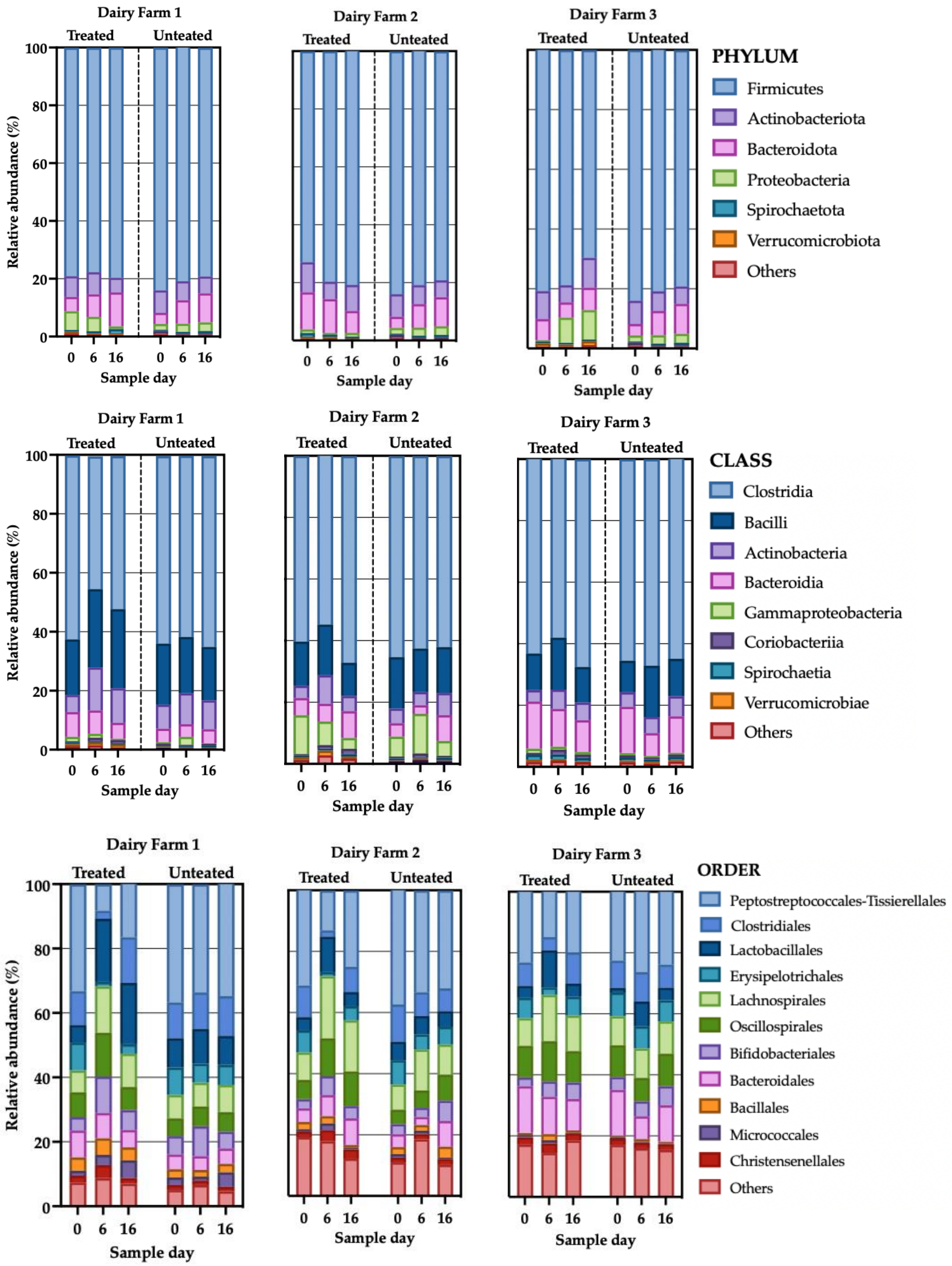

Figure S6. Fecal microbiome relative abundance phylogenetic profile by dairy farm, day, and treatment. Comparisons of relative abundance at the level of bacterial phylum, class, and order. Graphs were generated using GraphPad Prism version 10.0.2.

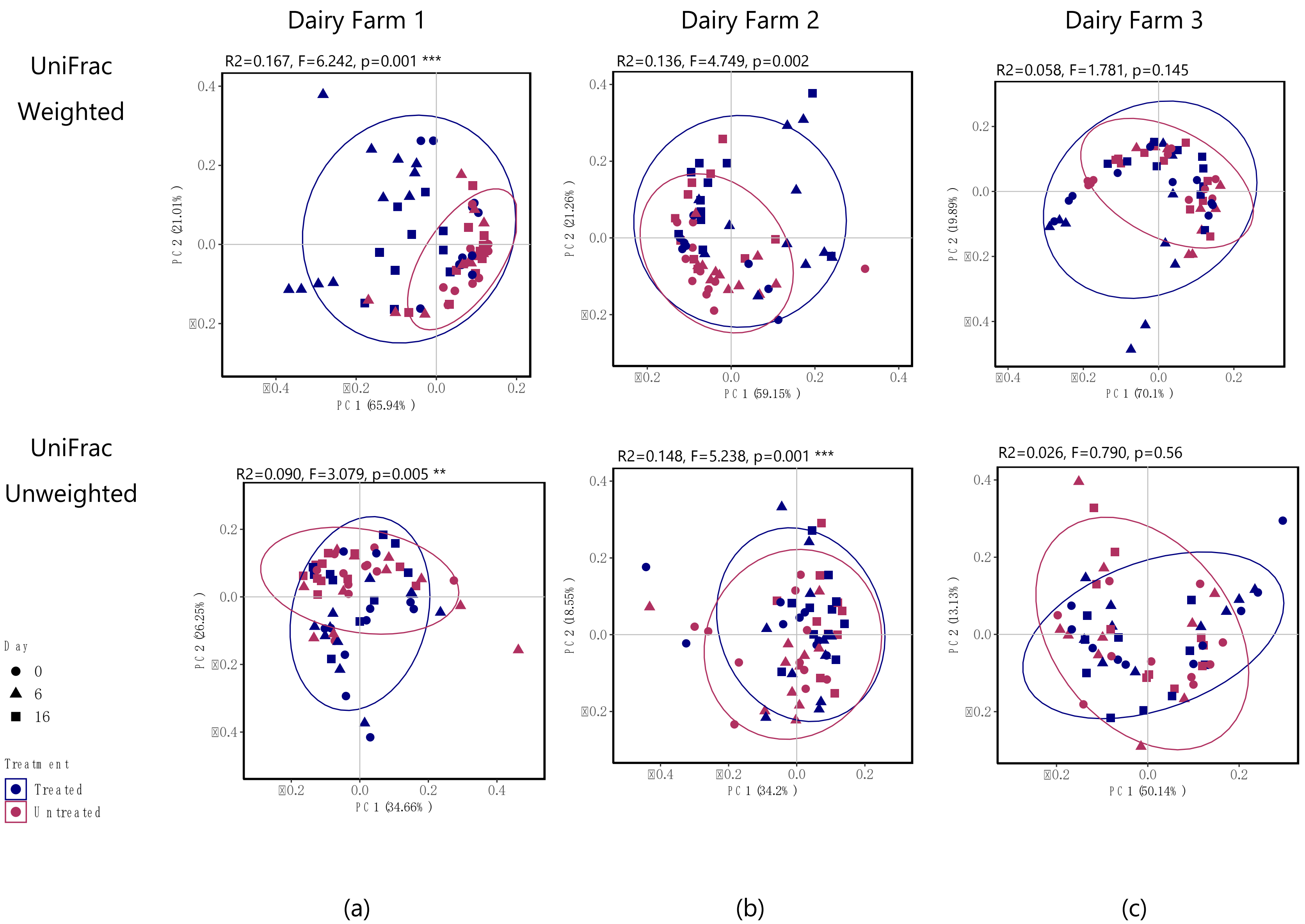

Figure S7. Fecal microbial beta-diversity represented in Principal Component Ordinal Analyses (PCoA) by dairy farm and clustered by day and treatment. UniFrac distances between bacterial community composition based on (top three) weighted (quantitative) and (bottom three) unweighted (qualitative). Figures were created using the Vegan package in R Studio.

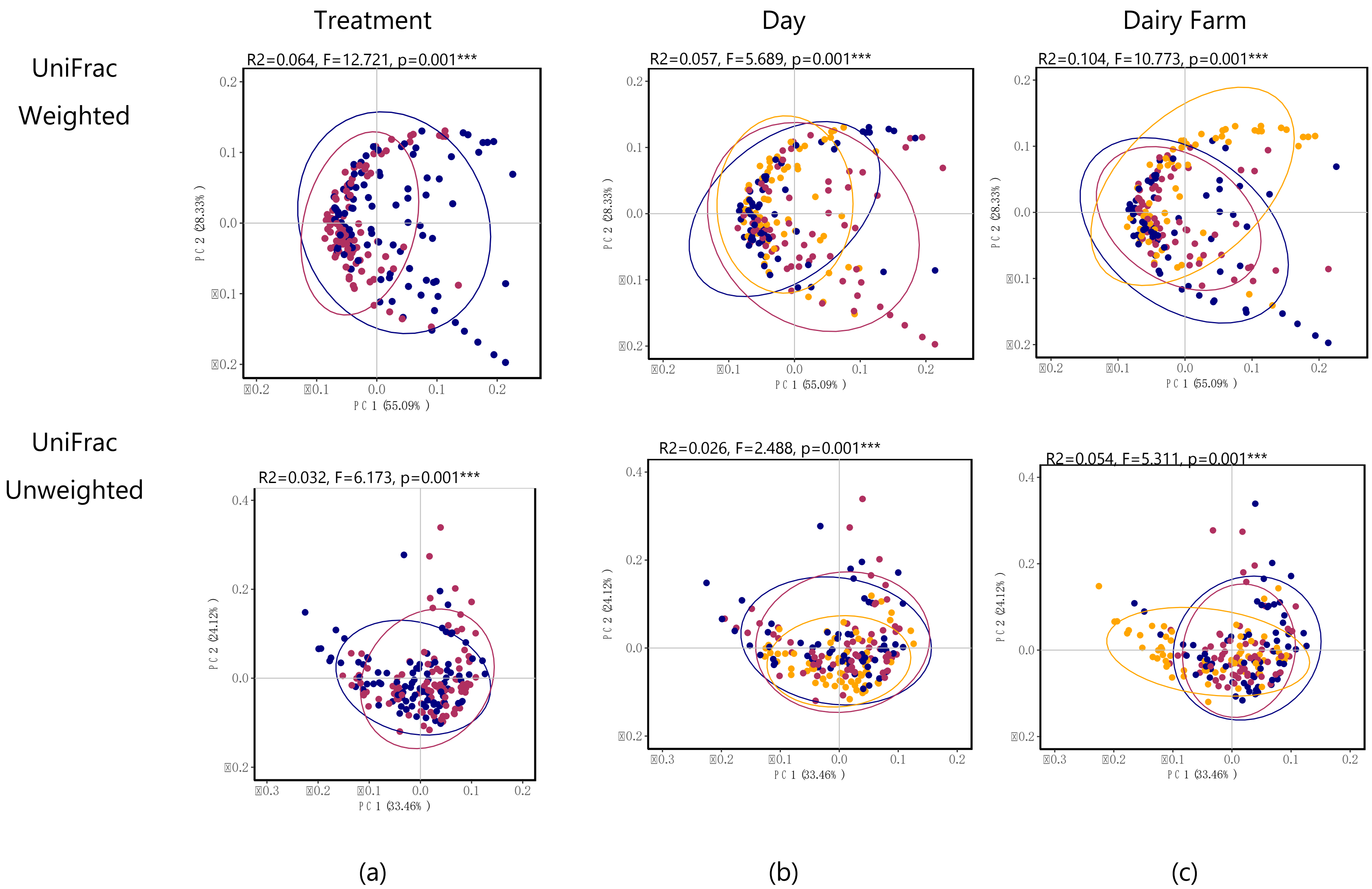

Figure S8. Fecal microbial beta-diversity represented in Principal Component Ordinal Analyses (PCoA) by dairy, day, and treatment. UniFrac distances between bacterial community composition based on (top three) weighted (quantitative) and (bottom three) unweighted (qualitative). Figures were created using the Vegan package in R Studio.
